# Train–test splits matter more for evaluation than for performance

**DOI:** 10.1186/s13321-026-01266-7

**Published:** 2026-08-04

**Authors:** Davide Crucitti, Adrián Mosquera Orgueira

**Affiliations:** 1 GrHeCo-Xen, Health Research Institute of Santiago (IDIS), University of Santiago de Compostela, Santiago de Compostela, Spain; 2 Department of Pharmacy, University of Santiago de Compostela, Santiago de Compostela, Spain; 3 Department of Hematology, University Hospital of Santiago de Compostela, Santiago de Compostela, Spain

**Keywords:** Train–test split, Model evaluation, Nemenyi test, Spearman, RMSE, BEDROC, Applicability domain, Drug discovery, LightGBM, QSAR

## Abstract

In machine learning for QSAR/QSPR, the choice of train–test splitting algorithm alters both a model’s realized performance and the accuracy with which that performance is estimated from the held-out test set. Prior work has established that structure-aware splits such as Kennard–Stone and SPXY produce optimistically biased internal estimates, but these characterizations typically examine one method family at a time. Here we quantify how fifteen splitting strategies from four method families differ across fifteen drug-discovery datasets on two criteria (realized external benchmark performance and performance-estimation bias), and further assess whether splitting strategy affects model-selection quality when multiple model classes are optimized simultaneously. A single, consistent pattern holds across all fifteen datasets and all eleven evaluation measures (RMSE, MAE, MedAE, $\hbox {R}^{2}$, Spearman ρ, Pearson r, Kendall τ, enrichment factor at 5/10/20%, and BEDROC): Kennard–Stone-type methods (KS, SPXY, MDKS, Morais) achieve the strongest external benchmark performance but produce the most optimistically biased internal estimates (median Spearman bias $\Delta \rho$ ≈ +0.17 to +0.22, SPXY most optimistic; median $\hbox {R}^{2}$ bias $\Delta \hbox {R}^{2}$ ≈ +0.26 to +0.41), diversity-based methods (OptiSim, Maximum/Minimum Dissimilarity) achieve the weakest benchmark performance, and cluster-shuffle variants, particularly k-means- and k-medoids-shuffle, provide the best-calibrated internal estimates (near-zero bias) while remaining competitive on benchmark performance. For every measure the differences in estimation bias between splitters greatly exceed the differences in benchmark performance (for Spearman ρ, a ≈0.20 bias gap versus a ≈0.02 benchmark gap), and the two criteria are inversely ranked (rank correlation ρ ≈ −0.70 to −0.83). For model selection among competing model classes, in contrast, no method achieves statistical separation and effect sizes are small, indicating that splitter choice has no demonstrated impact on which model class is ultimately selected. Splitter choice in drug-discovery QSAR therefore primarily affects the reliability of performance evaluation rather than realized model generalization. Cluster-shuffle splitting provides near-zero estimation bias while maintaining competitive external performance, making it the recommended default for offline model evaluation, where calibration is the primary concern. Because no splitter separates on model-selection quality, it is a sound default for model selection as well. Practitioners who wish to explicitly optimize model selection may use KS-type splitting to identify the best model configuration, then re-split with cluster-shuffle to obtain a calibrated performance estimate.

**Scientific contribution**

We provide the first head-to-head comparison of fifteen train–test splitting algorithms from four method families on a common drug-discovery regression benchmark, showing that their effect on the credibility of internal performance estimates is roughly an order of magnitude larger than their effect on realized model performance. We further show that this optimism is general across all eleven evaluation measures, give it a geometric explanation in terms of train–test distributional distance, and separate the splitting problem into two tasks, performance estimation and model selection, that have different optimal choices. On this basis we recommend cluster-shuffle splitting as a calibrated default; whereas prior work described the overoptimism of a single method family (Kennard–Stone), our comparison spans four families and all eleven measures and turns this into a concrete recommendation.

**Supplementary Information:**

The online version contains supplementary material available at https://doi.org/10.1186/s13321-026-01266-7.

## Introduction

In supervised machine learning for QSAR/QSPR, reliable offline evaluation depends critically on how available data are partitioned into training and test subsets. Ideally, the test set should represent the compounds a deployed model will encounter: future, unseen molecules that may differ systematically from the training set. In practice, the test set is always drawn from the same corpus as the training data, making the split strategy a key determinant of whether internal evaluation is a faithful proxy for prospective performance.

Different splitting strategies impose different degrees of train–test dissimilarity, and this dissimilarity controls two distinct and often competing outcomes. First, it affects the training distribution and hence the model’s realized generalization: methods that concentrate structurally diverse or distant compounds in the test set tend to leave the model a less representative training set, potentially depressing external benchmark performance. Second, it affects evaluation credibility, the degree to which a measure computed on the internal test set accurately reflects the model’s performance on the real prospective challenge. Methods that create artificially easy test sets (i.e., sets that are internally similar to the training data) produce optimistic internal estimates that overstate prospective performance, while methods that create harder test sets tend to produce more conservative estimates.

The literature on these effects is substantial. Kennard–Stone (KS) [1] and its extensions (SPXY [2], MDKS [3], and Morais–Lima–Martin [4]) select test samples that span the descriptor space uniformly. This maximizes the training set’s coverage of the descriptor space but leaves the internal test set as a representative subsample of the development pool rather than a challenging hold-out. The consequences are well documented: KS and SPXY produce overoptimistic performance estimates, particularly when the test fraction is large [5–9]. Data-leakage effects from related methods have also been identified [10]. Diversity-based selection methods such as OptiSim [11], Minimum Dissimilarity, and Maximum Dissimilarity take the opposite approach, maximizing train–test separation; prior work suggests these produce more conservative estimates. Clustering-based splits [12] occupy an intermediate position.

The optimism of structure-aware splits is well established, but most quantitative characterizations come from spectroscopic and analytical-chemistry data and typically examine one method family at a time [5, 13]. Less settled is how the major method families compare directly, on the same footing and across many evaluation measures, in the drug-discovery regression setting, and in particular how large the resulting differences in estimation error are relative to the accompanying differences in benchmark performance.

Two further aspects have received less systematic attention. First, internal test evaluation serves not only as a performance estimator but as a model-selection guide: practitioners use internal test scores to choose among competing configurations (algorithm class, hyperparameters), and it is unclear which splitting strategy produces internal rankings most correlated with external benchmark performance. Second, the applicability domain (AD) of a model determines whether a predicted value for a test compound is trustworthy; a training set whose AD barely covers the external benchmark means prospective performance is evaluated largely on out-of-domain compounds, reducing the interpretability of those measures regardless of their numerical value.

Using fifteen drug-discovery datasets with an externally defined temporal benchmark, we ask: (i) which splitting strategies yield higher external benchmark performance, (ii) which strategies most accurately estimate that performance using internal test data, (iii) which strategies produce models with higher applicability-domain coverage of the external benchmark, and (iv) which strategies best support model selection across competing model configurations.

## Methods

### Datasets

We analyzed fifteen anonymized drug-discovery regression datasets from the publicly released Merck benchmark [14, 15]. Table 1 summarizes the 15 datasets.

**Table 1:** Dataset descriptions

Dataset	Endpoint type	Development pool	External benchmark	Total
3A4	CYP activity	37,241	12,338	49,579
CB1	Activity	8,716	2,907	11,623
DPP4	Activity	6,148	2,045	8,193
HIVINT	Activity	1,815	598	2,413
HIVPROT	Activity	3,212	1,072	4,284
LOGD	ADME	37,388	12,406	49,794
METAB	ADME	1,569	523	2,092
NK1	Activity	9,965	3,335	13,300
OX1	Activity	5,351	1,769	7,120
OX2	Activity	11,151	3,704	14,855
PGP	ADME	6,399	2,093	8,492
PPB	ADME	8,651	2,899	11,550
RAT_F	ADME	6,105	1,707	7,812
TDI	Activity	4,165	1,382	5,547
THROMBIN	Activity	5,059	1,698	6,757

Development pool = earlier-tested compounds (used exclusively for internal model training and evaluation). External benchmark = later-tested compounds (temporal holdout, held out throughout; used only to compute external performance measures)

Dataset sizes span approximately 24-fold, from METAB (2,092 compounds) to LOGD (49,794 compounds), providing meaningful variation in the regime where splitting artifacts are known to scale with dataset size [13]. Molecular structures are not available in the public release; we use the provided precomputed atom-pair and donor–acceptor–pair descriptors. No additional preprocessing was performed.

### Study design

Each dataset includes an externally defined temporal holdout provided by Merck. We treat this holdout as an immutable external benchmark: it is never used for model training or splitting-algorithm selection, only for final performance evaluation. All remaining data form the development pool.

Within the development pool, we apply 5 repeats × 5-fold outer cross-validation, producing 25 partially overlapping CV base sets per dataset (each comprising ≈80% of the development pool). This repeated structure reduces variance due to any single partition and provides 25 evaluation replicates per (dataset, splitter) pair. Because the repeated-CV base sets overlap (~80% shared compounds), these replicates are correlated rather than independent; accordingly, all across-method inference is performed at the level of the 15 datasets, with the per-replicate values only averaged to a single per-(dataset, splitter) score. Within each CV base set, each splitting algorithm partitions data into an internal train subset (≈80% of the CV base set) and an internal test subset (≈20%). A model is fitted on the internal train subset only and evaluated on both the internal test subset and the external benchmark.

To reduce dependence on any single descriptor subset, each fit is repeated with ten different feature-subsampling seeds controlling LightGBM’s internal feature subsampling (feature_fraction = 0.8, applied per boosting iteration), and the measures are averaged.

### Splitting algorithms

We compared fifteen splitting strategies across four families.

1. **Kennard–Stone-type methods** select the training set to span the descriptor space uniformly, leaving interior (low-dissimilarity) compounds in the test set. We include four variants: Kennard–Stone (KS) [1], SPXY [2] (which jointly maximizes dissimilarity in both descriptor and activity space), MDKS [3], and Morais–Lima–Martin [4]. Because the test set is composed of compounds close to the training set, these methods are known to generate optimistic performance estimates [5–7].
2. **Diversity-based methods** maximize train–test dissimilarity. OptiSim [11] selects test compounds from a pool of maximally diverse candidates; Maximum Dissimilarity (MaxDiss) greedily selects the test compound furthest from the training set; Minimum Dissimilarity (MinDiss) ensures every test compound exceeds a minimum distance from its nearest training neighbor. These methods create structurally remote test sets; prior work expects conservative internal estimates, although in our benchmark their estimates remain mildly optimistic.
3. **Clustering-based methods** [12] partition the descriptor space into clusters and assign compounds to train/test accordingly. Two assignment policies are compared: - *Cluster-shuffle*: entire clusters are assigned to either train or test, so no two compounds from the same cluster appear on opposite sides of the split. - *Cluster-stratified*: compounds are sampled proportionally from each cluster into both subsets, preserving the cluster distribution on both sides.

Three base clustering algorithms are used: hierarchical agglomerative clustering (HAC), k-means, and k-medoids, each with 10 clusters. These are combined with both policies, plus sphere-exclusion (Taylor–Butina) clustering [16] in shuffle mode only (variable cluster sizes make proportional stratification impractical), giving seven clustering splitters in total.

4. **Random split:** uniform random assignment without stratification, serving as the primary baseline.

### Splitting-assessment experiment (fixed model)

For the splitting assessment experiment, all models use LightGBM with fixed hyperparameters: n_estimators = 500, learning_rate = 0.05, feature_fraction = 0.8 [17]. We hold the model class and configuration constant to isolate splitting effects from modeling effects. For each (dataset, CV base set, splitting algorithm) combination, we train 10 independent LightGBM models on the internal train subset, each with a different random seed that controls the feature-fraction sampling. Reported measures are the average over these 10 runs, reducing Monte Carlo variance in the feature-fraction randomness. All 15 datasets are analyzed at full development-pool size.

The Mahalanobis Distribution Alignment Score (MDAS) [18] quantifies the distributional distance between each split’s internal test set (or external benchmark) and the training set. MDAS is a symmetric measure computed from the pooled train+test descriptor covariance, capturing both mean and covariance differences; the covariance is inverted via Cholesky decomposition with a ridge-regularized fallback for rank-deficient cases.

### Sensitivity analysis: small-dataset regime

To probe robustness in the small-data regime, we conducted two subsampling experiments in which every outer CV base set was capped before splitting. The primary analysis caps at 150 compounds (“Sub-150 analysis”), chosen because datasets of this size are representative of real-world ADMET screening campaigns and early-stage drug-discovery assays, where experimental budgets are tight and splitting artefacts are most consequential for decision-making. A secondary analysis capping at 1,000 compounds (“Sub-1000 analysis”) is reported in Additional file 1 (Figs. S15–S17) for comparison across the size spectrum. All other aspects of the pipeline (splitting algorithms, modeling, evaluation measures, repeated CV structure) are identical to the main analysis.

### Model-selection experiment

To assess which splitting strategy most reliably identifies the best model when multiple model classes compete, we run a separate champion-selection experiment using the same CV base sets. For each (dataset, CV base set, splitting algorithm), we apply Tree-structured Parzen Estimator (TPE) hyperparameter optimization [19] with three-round successive halving across five model classes: LightGBM (gradient-boosted trees), Random Forest, KNN Regressor, Support Vector Regressor (ε-SVR, RBF kernel), and Elastic Net. Tuned hyperparameters for each class are listed in Additional file 1: Table S2.

The successive-halving schedule allocates up to 35 TPE trials to the eventual winner: Round 1 runs all five classes for 5 trials each with 2-fold inner CV (top 2 advance); Round 2 runs the two survivors for 15 trials each with 5-fold inner CV (top 1 advances); Round 3 runs the single survivor for the remaining 15 trials with 5-fold inner CV. When the training set contains more than 5,000 compounds, TPE trials are performed on a random subsample of 5,000 compounds to keep SVR computationally tractable; final retraining of the selected model always uses the full inner-train set.

All five model classes are retrained on the full internal train subset after model selection is complete, so that their predictions on the internal test and external benchmark are recorded.

### Evaluation measures

We report eleven measures covering complementary aspects of regression and virtual-screening quality:

- Error measures (lower is better): RMSE, MAE, MedAE (median absolute error, more robust to outliers than MAE)
- Explained variance (higher is better): $\hbox {R}^{2}$
- Correlation measures (higher is better): Spearman ρ and Kendall τ (rank-based), and Pearson r (linear correlation)
- Enrichment measures (higher is better): Enrichment factor at the top 5%, 10%, and 20% of the predicted ranking (EF-5%, EF-10%, EF-20%), and BEDROC. For each EF, “actives” are the true top-*k*% compounds by measured activity; EF = (hits/n_selected)/(n_actives/*n*), where EF = 1 indicates random ranking and EF = 1/*k* is the theoretical maximum. BEDROC [20] (α = 20, active fraction = top 20% by measured activity) extends EF by weighting early-ranked hits exponentially more heavily, making it sensitive to recovery of the highest-scoring actives.

### Performance-estimation bias and evaluation credibility

For each dataset × algorithm × CV base set, the benchmark gap is:

$$\begin{aligned} \Delta = S_{\text {internal test}} - S_{\text {benchmark}} \end{aligned}$$

where S is computed on an oriented scale (higher always better). Δ>0 indicates optimism (the internal estimate overstates the model’s true prospective performance); Δ<0 indicates pessimism. We report mean Δ (signed bias) and mean |Δ| (estimation error, the unsigned magnitude) as measures of evaluation credibility: a split that yields a small |Δ| provides credible, well-calibrated internal estimates of prospective performance.

### Applicability domain

We fit a k-nearest-neighbor distance-based applicability domain on the internal train subset (k = 5, threshold z = 1.5 standard deviations above the mean training-set NN distance) and compute the fraction of external benchmark compounds that fall within the AD (coverage). We further compare external benchmark performance on in-domain versus out-of-domain benchmark compounds, and report the internal test estimate as a reference. AD coverage bears on evaluation interpretability: predictions for in-domain compounds are more trustworthy than those for out-of-domain compounds, so scores computed on a benchmark with higher AD coverage are more interpretable.

### Model selection quality

For each CV base set, the model-selection experiment provides, for each splitting algorithm, a selected model and four runner-up models, each with their best-found hyperparameters, an inner-CV score, and an external benchmark score. We assess model-selection quality using two complementary measures:

1. Selection advantage: the external benchmark Spearman ρ of the internally selected model minus the external benchmark Spearman ρ of the best alternative model configuration. Positive values indicate that the internally selected model outperforms the best alternative externally; negative values indicate that a different model would have generalized better.
2. Selection hit rate: the fraction of folds in which the internally selected model is also the externally best-performing model. A higher hit rate means the splitting strategy more reliably identifies the best-generalizing model.

### Statistical analysis

Across-dataset inference: For each dataset, algorithms are ranked on each target measure (average over 25 CV base sets); ranks are averaged over the 15 datasets. This rank-based aggregation is essential because several measures, in particular the error measures RMSE and MAE, are expressed in each dataset’s own activity units and are therefore not comparable across datasets as raw values. We use the Friedman test with Nemenyi post-hoc comparisons [21], following the approach recommended for benchmarking multiple algorithms across multiple datasets. A non-parametric rank-based procedure is preferred: measure distributions are heterogeneous across datasets, normality cannot be assumed, and the correlated CV replicates make parametric variance estimation unreliable. The Friedman test assesses whether mean ranks differ significantly across splitters; upon rejection (all cases, p < 0.001), the Nemenyi post-hoc test identifies statistically indistinguishable groups at α = 0.05. The Nemenyi critical difference (CD) is the minimum rank difference required for two algorithms to be distinguished at the chosen α; pairs connected by a horizontal bar in CD diagrams [21] are statistically indistinguishable at this threshold. The Nemenyi test is appropriate for the complete block design of our benchmark (all 15 splitters × all 15 datasets).

Effect sizes: We report median and interquartile range of per-dataset measure values alongside CD diagrams to convey practical magnitude independent of statistical significance thresholds.

### Multicriteria decision analysis

To complement the two-objective (Pareto) analysis with a single overall ranking, we applied two established multicriteria decision-analysis (MCDA) methods, TOPSIS [22] and the ratio-system MOORA [23], via the JMcDM implementation [24]. Each takes a decision matrix of alternatives × criteria with per-criterion weights and benefit/cost directions, applies vector normalization, and returns a scalar score per alternative (TOPSIS: closeness to the ideal solution, in [0, 1]; MOORA: a weighted benefit-minus-cost value). Because raw measure values are not comparable across datasets (above), we ran each MCDA within each dataset, taking the fifteen splitters as alternatives and the eleven measures as equally-weighted criteria (benefit or cost according to each measure’s direction), and averaged the per-dataset ranks across the fifteen datasets, as for the Nemenyi analysis. We evaluate three criterion sets: benchmark performance (the external-benchmark scores on the eleven measures), estimation credibility (the absolute estimation gaps |Δ| on the eleven measures, all cost criteria), and their combination (all twenty-two criteria with equal weights). TOPSIS and MOORA agreed closely, so we report TOPSIS in the main text.

## Results

### Cross-measure overview

**Fig. 1:**
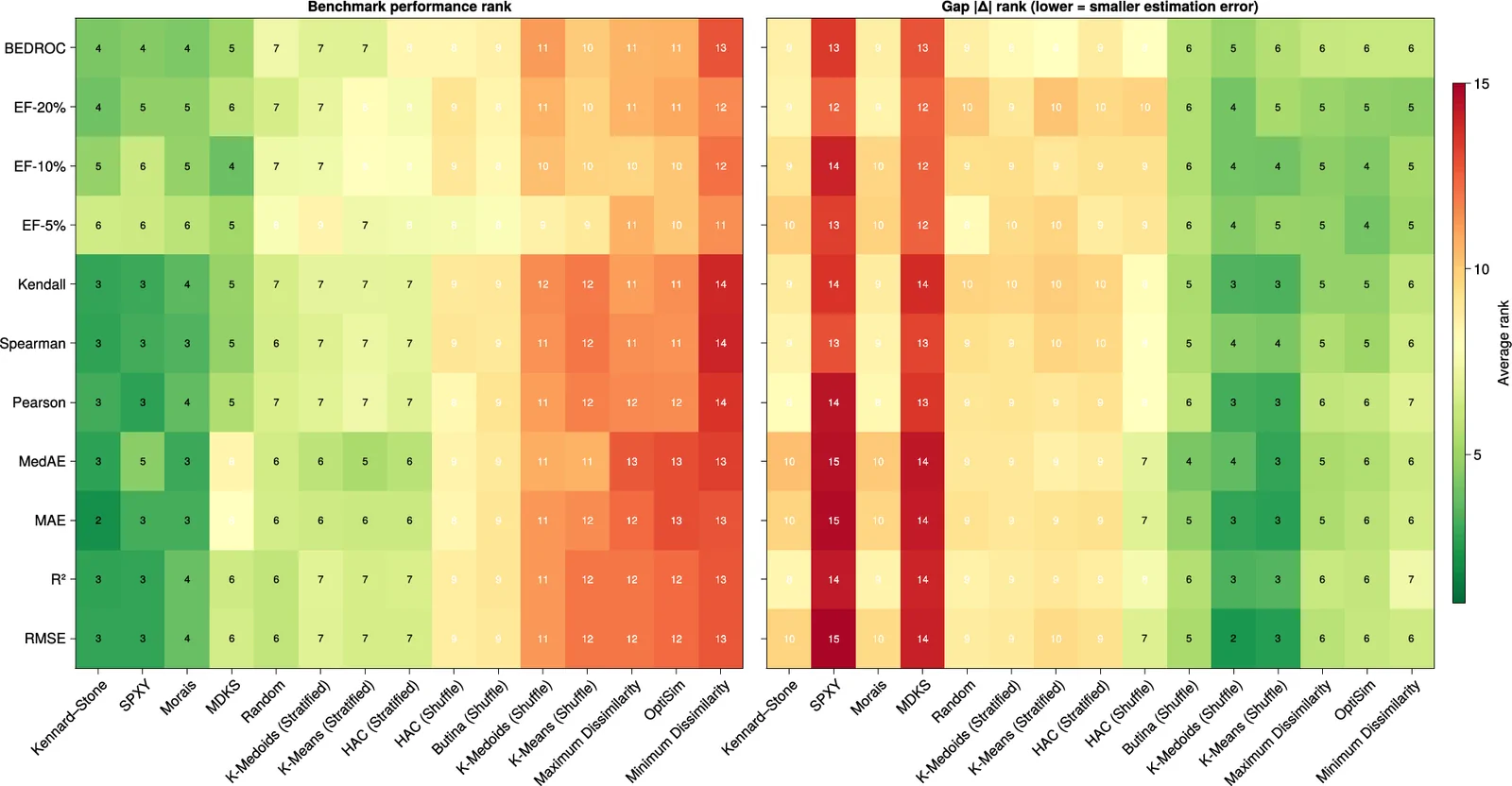
Nemenyi average rank heatmap across all eleven measures. Each cell is a splitter’s average rank for that measure (ranked within each dataset, then averaged across the fifteen datasets). Left panel: benchmark performance (green = rank 1, best); right panel: estimation gap |Δ| (green = rank 1, best calibrated)

Figure 1 presents the complete picture: a heatmap of Nemenyi average ranks across all fifteen splitting strategies and eleven measures, for benchmark performance (left panel) and estimation gap |Δ| (right panel). KS-type methods dominate the benchmark panel but rank last on calibration; k-means and k-medoids-shuffle lead on calibration while maintaining intermediate benchmark ranks. The following sections examine Spearman ρ as the primary worked example; RMSE, $\hbox {R}^{2}$, and enrichment confirm the same pattern throughout.

Additional file 1: Figure S9 shows the family-level mean rank bar chart aggregated across all methods and measures, confirming that cluster-shuffle is the only family achieving competitive benchmark performance and the best calibration rank simultaneously.

### External benchmark performance

#### Spearman rank correlation

**Fig. 2:**
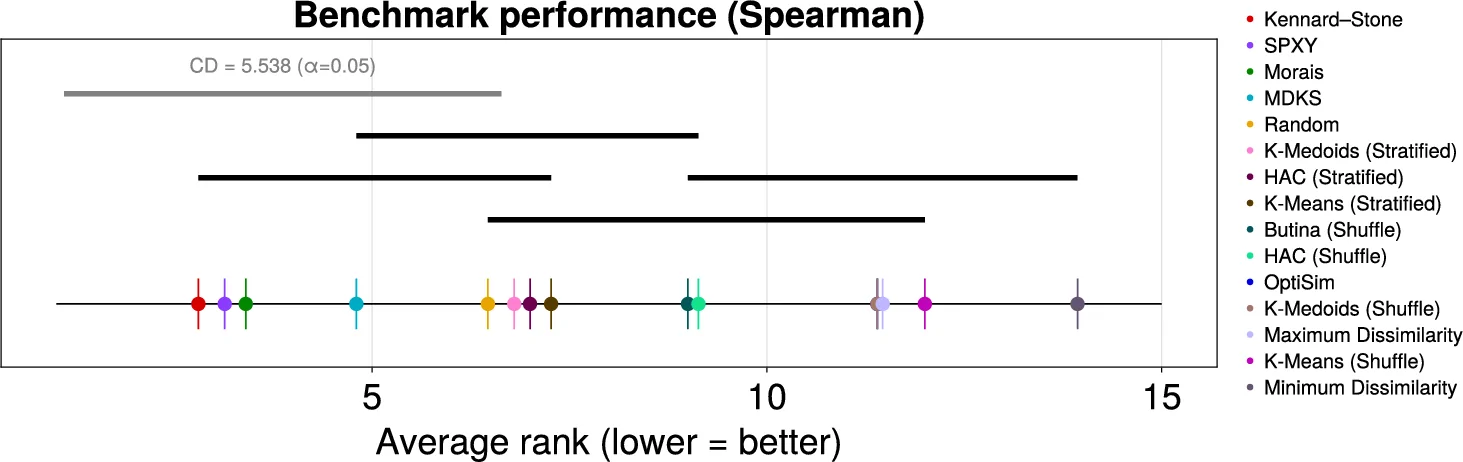
Nemenyi CD diagram for external benchmark Spearman ρ (CD = 5.538, α = 0.05; lower rank = better)

Figure 2 shows the Nemenyi CD diagram for benchmark Spearman ρ (average rank across 15 datasets; lower rank = higher ρ; CD = 5.538 at α = 0.05). Three performance tiers are visible. KS-type methods (SPXY, KS, MDKS, Morais; ranks 1–4) form the top tier. Diversity-based methods (OptiSim, MinDiss, MaxDiss; ranks 12–15) form the bottom tier. An intermediate tier (Random, cluster-stratified variants, HAC-shuffle, Butina-shuffle, and the remaining cluster-shuffle methods) occupies ranks 5–12. KS-type methods are statistically distinguishable from the lowest-ranked diversity methods (rank gap exceeds the CD); adjacent tiers overlap under the CD bar, with no significant pairwise separation between neighboring groups.

Spearman ρ medians differ by $\Delta \rho$ ≈ 0.09 between the best and worst methods (Additional file 1: Fig. S1); within-method variance across datasets substantially exceeds between-method differences, confirming that dataset identity dominates over splitter choice for realized benchmark performance. The same three-tier ordering holds across all eleven measures (Additional file 1: Fig. S2; summarized in Fig. 1): KS-type methods consistently rank highest, diversity-based methods lowest, and cluster-shuffle methods intermediate.

### Performance-estimation bias and evaluation credibility

#### Spearman estimation error

**Fig. 3:**
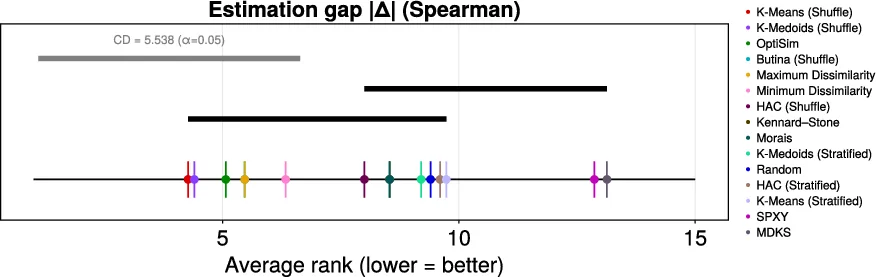
Nemenyi CD diagram for Spearman estimation error |ΔSpearman| (CD = 5.538, α = 0.05; lower rank = better calibrated)

Figure 3 shows the CD diagram for absolute estimation error |ΔSpearman|. The family ranking inverts relative to Fig. 2. k-means-shuffle and k-medoids-shuffle are the best-calibrated methods (ranks 1–2), and together with diversity methods and the remaining cluster-shuffle variants (ranks 3–7) form the well-calibrated tier. SPXY and MDKS form the worst-calibrated tier (ranks 14–15) and are statistically distinguishable from the well-calibrated group (rank gap exceeds the CD). KS, Morais, cluster-stratified variants, and Random occupy an intermediate tier (ranks 8–13) indistinguishable from both extremes under the CD bar.

**Fig. 4:**
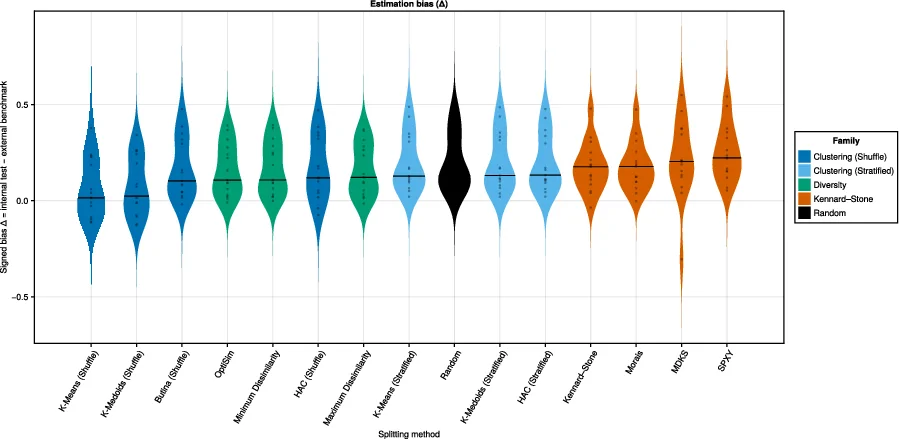
Signed Spearman estimation bias Δ = ρ_internal_test – ρ_benchmark across all (dataset, CV base set) combinations, sorted from best-calibrated (left) to worst (right). Dashed line: Δ = 0 (perfect calibration)

Figure 4 shows the signed estimation bias for each splitter, sorted from best-calibrated (left) to worst (right). Every method is optimistic (Δ > 0), but by very different amounts: k-means- and k-medoids-shuffle sit near zero (median Δ ≈ +0.02), while SPXY reaches median Δ ≈ +0.22, with a clear gradient across the remaining methods. The violin widths reflect substantial between-dataset variability: within-method spread often exceeds between-method differences, reinforcing that dataset identity is a stronger determinant of absolute bias magnitude than splitter choice.

Estimation-error differences far exceed benchmark-performance differences: the bias gap between SPXY (Δ ≈ +0.22) and k-means-shuffle (Δ ≈ +0.02) is approximately 0.20, roughly ten times the benchmark Spearman difference between the same two methods (≈0.02). Across the full method set, the between-family bias spread (≈0.22) exceeds the between-family benchmark-performance spread (≈0.09) by approximately 2.5-fold.

**Fig. 5:**
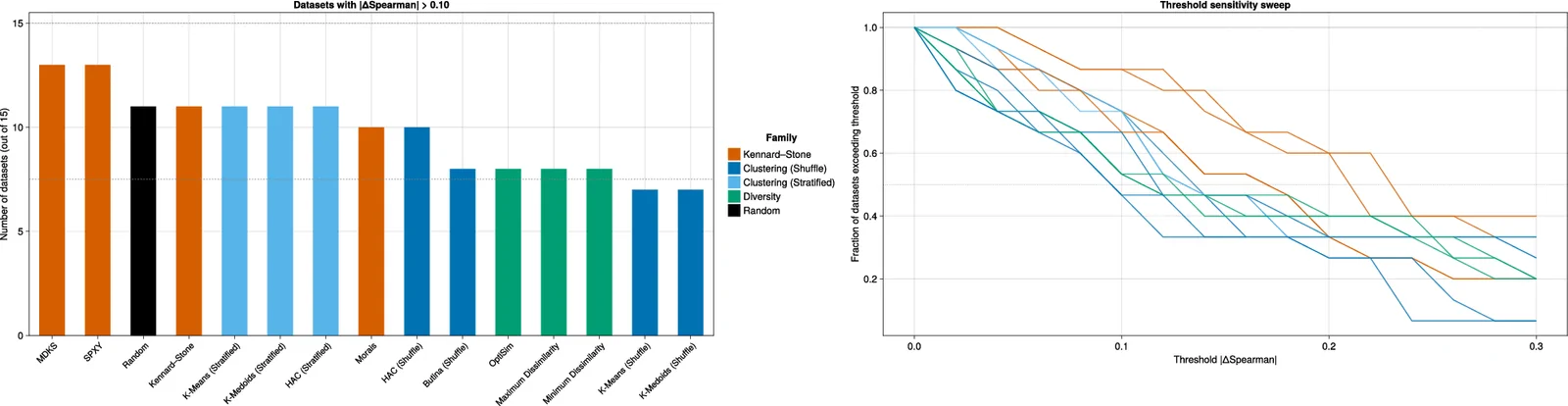
Left: number of datasets (out of 15) in which |ΔSpearman| exceeds 0.10, colored by method family. Right: fraction of datasets exceeding each threshold from 0 to 0.30

Figure 5 shows this holds across datasets: SPXY and MDKS exceed $\bar{\Delta }$Spearman$\bar{>}$ 0.10 in 13 out of 15 datasets, while k-means- and k-medoids-shuffle do so in 7 out of 15, but with near-zero median bias (Δ ≈ +0.02), their exceedances reflect dataset-level variance rather than systematic optimism. Pessimistic bias (Δ < 0) was rare for KS-type methods, confirming their optimism is nearly universal; cluster-shuffle methods have symmetric scatter around zero and no consistent direction. The same family ordering holds across all eleven measures (Additional file 1: Fig. S3; Figs. S18–S33 for individual per-measure CD diagrams; summarized in Fig. 1), with $\hbox {R}^{2}$ bias ranging from $\Delta \hbox {R}^{2}$ ≈ +0.41 for SPXY to ≈+0.03 for k-means/k-medoids-shuffle as an illustrative example. This finding directly confirms and quantifies the optimism of KS-type methods documented in the analytical chemistry and chemometrics literature [5–8].

### Performance–estimate inversion

**Fig. 6:**
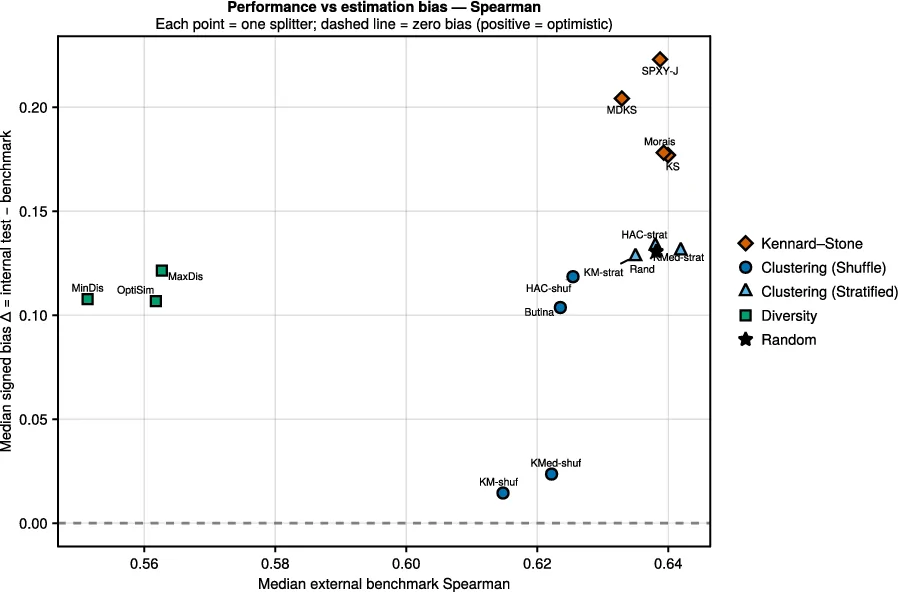
Median external benchmark Spearman ρ (x-axis) vs. median signed estimation bias Δ (y-axis), one point per splitting method. Dashed line: zero bias. The ideal method sits bottom-right

The benchmark-performance and calibration rankings are negatively correlated (rank correlation ρ = −0.73; Additional file 1: Fig. S4): methods that rank best on benchmark rank worst on calibration. Figure 6 makes this concrete: k-means- and k-medoids-shuffle occupy the Pareto-efficient position (high benchmark performance, near-zero bias); KS-type methods lead on benchmark at the cost of large bias; diversity methods score worst on benchmark performance but carry intermediate bias (~0.11); KS-type methods carry the largest bias (0.17–0.22). No single splitter wins on both criteria simultaneously; the $\hbox {R}^{2}$ analogue of this trade-off is shown in Additional file 1: Fig. S5.

### Multicriteria decision analysis

**Fig. 7:**
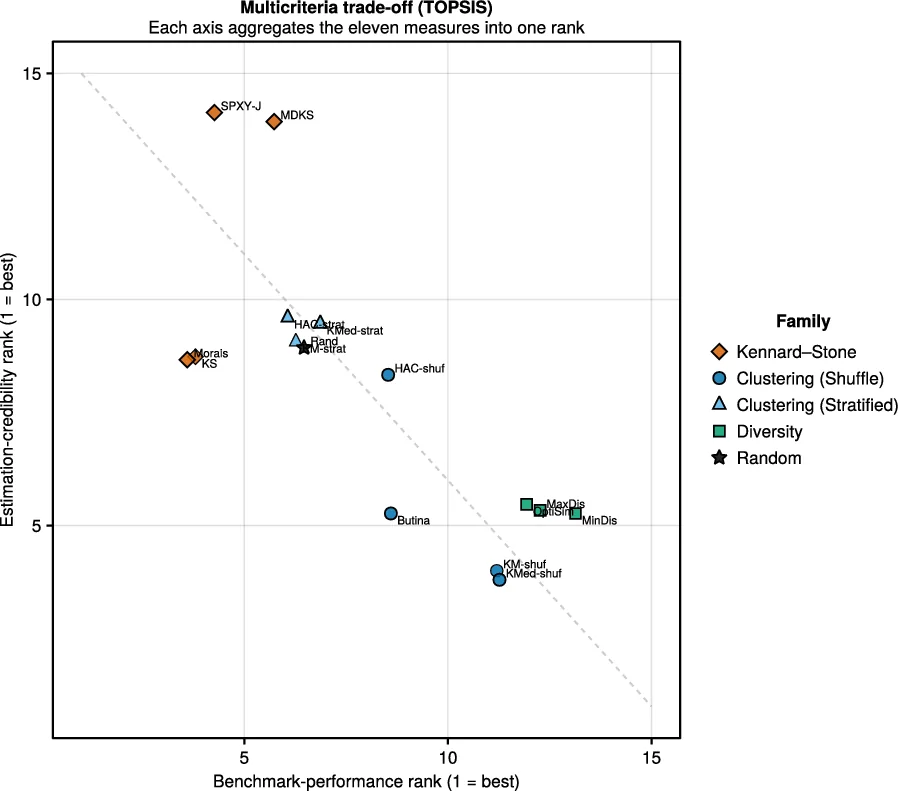
Multicriteria trade-off between benchmark performance and estimation credibility (TOPSIS). Each rank is a single TOPSIS score that aggregates all eleven measures as criteria, with 1 best on each axis. The dashed line marks a perfect inversion between the two rankings

Applying TOPSIS and MOORA with the eleven measures as criteria recovers the two-objective trade-off and condenses it into a single overall ranking (full per-criterion ranks for both methods in Additional file 1: Tables S4 and S5). Ranked on benchmark performance, the Kennard–Stone family is best (TOPSIS mean ranks: Morais 3.6, Kennard–Stone 3.8, SPXY 4.3) and diversity methods worst (11.9–13.1). Ranked on estimation credibility, the ordering inverts: cluster-shuffle methods lead (k-medoids-shuffle 3.8, k-means-shuffle 4.0), and the Kennard–Stone methods rank last (MDKS 13.9, SPXY 14.1). The combined ranking, which weights the two objectives equally, therefore rewards methods that are credible without being weak on performance: k-medoids-shuffle (3.8), k-means-shuffle (3.9) and Butina-shuffle (4.9) take the top three places, while SPXY (13.6) and MDKS (13.7) are last. This trade-off generalizes the single-measure inversion of Fig. 6 to all eleven measures: as Fig. 7 shows, no splitter ranks well on both performance and credibility. MOORA reproduces the combined ordering almost exactly (Spearman ρ = 0.98 between the two methods’ combined ranks). The ranking is also robust to the measures’ redundancy: weighting each construct (error, explained variance, correlation, enrichment) equally, rather than each of the correlated measures, leaves the combined ranking essentially unchanged (Spearman ρ = 0.97). The MCDA delivers a single overall ranking, but which method comes first depends on how performance and credibility are weighted; cluster-shuffle is the strategy that performs best when the two objectives are weighted equally.

### MDAS analysis

**Fig. 8:**
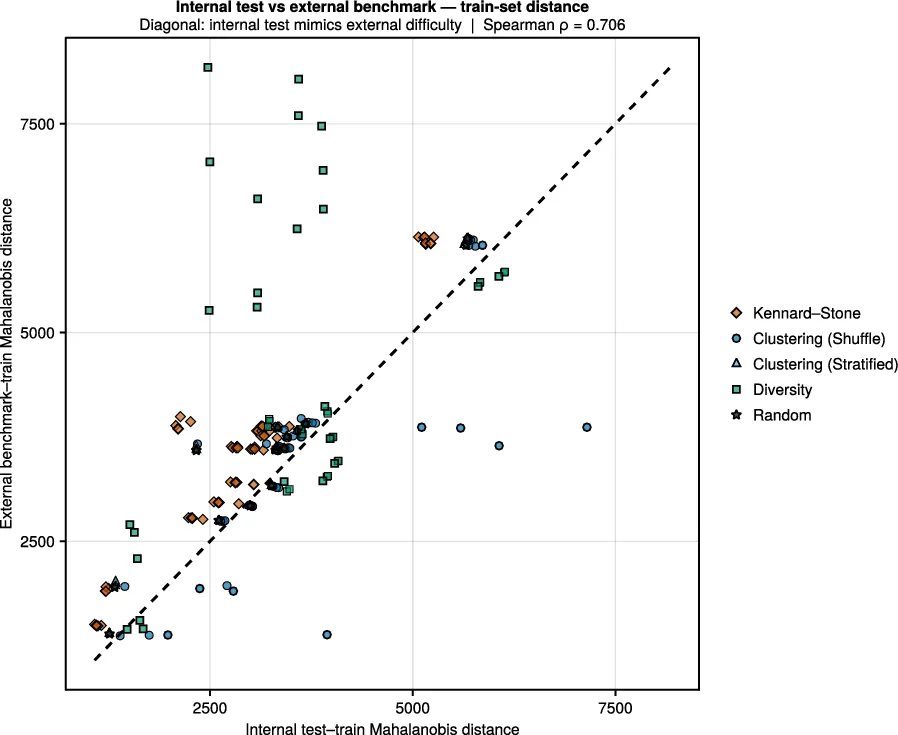
Internal test–train MDAS (x-axis) vs. external benchmark–train MDAS (y-axis), one point per (splitter, dataset) combination. Points on the diagonal have equal distributional distance; above the diagonal indicates the internal test is closer to training than the external benchmark

Figure 8 plots the MDAS from the internal test set to the training set (x-axis) against the MDAS from the external benchmark to the training set (y-axis), for each (splitter, dataset) combination (Spearman ρ = 0.71 across all pairs). KS-type methods sit above the diagonal with low internal MDAS: their internal test sets are much closer to training than the external benchmark, directly explaining their optimistic bias. Diversity methods also lie above the diagonal with similar or lower internal MDAS to KS (their diverse training sets cover internal chemical space broadly, keeping internal test performance moderate), but the external benchmark–train MDAS is substantially higher, placing them far above the diagonal. Cluster-shuffle methods are the rightmost group, sitting at or right of the diagonal: they create internal test sets as structurally remote from training as the external benchmark, or more so, which explains their near-zero bias.

Additional file 1: Figure S10 summarizes this relationship at the per-splitter level: methods whose external MDAS is close to their internal MDAS show smaller |ΔSpearman|, confirming that geometric split quality is predictive of calibration quality. Full 15×15 Nemenyi pairwise p-value matrices are provided as Additional file 1: Figs. S11 and S12, and the nearest-neighbor test–train distance distributions underlying this geometry as Additional file 1: Fig. S7.

### Applicability domain coverage

**Fig. 9:**
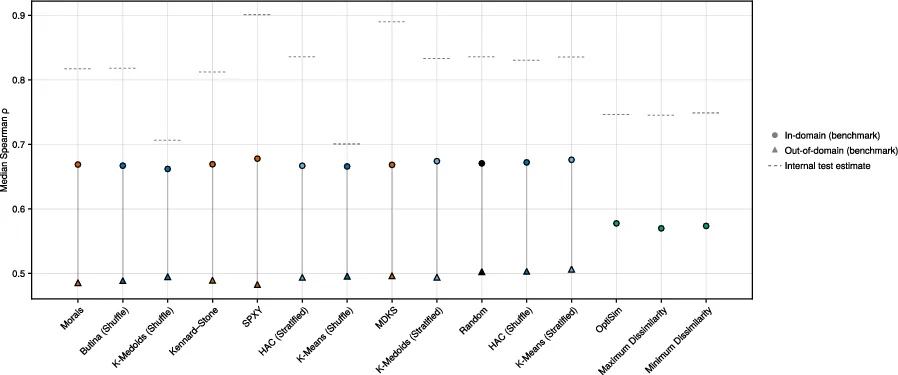
Median external benchmark Spearman ρ by splitting method, stratified by applicability-domain membership (circles = in-domain; triangles = out-of-domain). Dashed line: internal test estimate

Figure 9 stratifies external benchmark Spearman ρ by AD membership (in-domain vs. out-of-domain) for each splitter. For KS-type methods the in-domain benchmark half scores substantially higher than the out-of-domain half, yet the internal estimate (dashed) sits above even the in-domain score, confirming that KS inflates its internal estimates beyond what easy in-domain compounds alone can explain. Cluster-shuffle methods show a similar in-domain vs. out-of-domain gap to other families (~0.17), but their internal estimate (dashed) aligns with the overall benchmark level rather than sitting above it, consistent with their test sets being as structurally challenging as the temporal holdout. Diversity-based methods achieve the highest AD coverage; coverage fractions across splitters are shown in Additional file 1: Fig. S8.

### Model selection quality

**Fig. 10:**
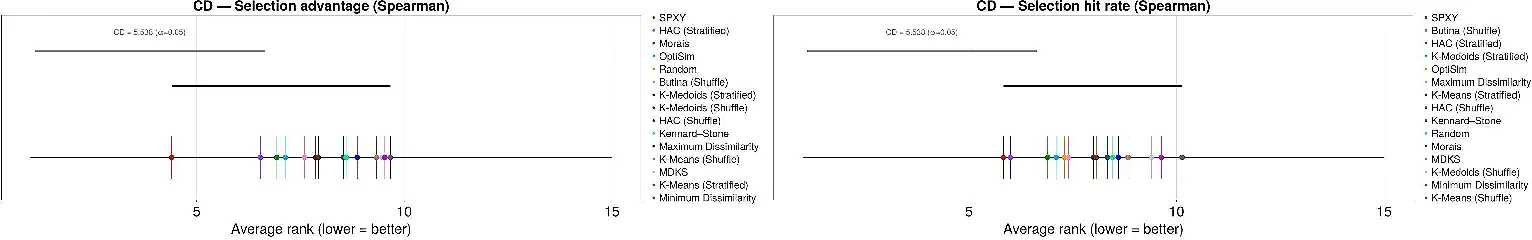
Model-selection quality (CD = 5.538, α = 0.05; lower rank = better). Left: selection advantage. Right: selection hit rate

Figure 10 reveals an inversion: calibration quality and model-selection quality point in opposite directions. SPXY, the worst-calibrated splitter by estimation error, ranks among the top on both selection advantage and hit rate; k-means-shuffle, the best-calibrated splitter, ranks last on both. KS-type methods generally lead model selection while cluster-shuffle variants occupy the lower half of the table. The likely mechanism is that geometrically easy interior test sets (KS-type) preserve relative model rankings even while inflating absolute estimates: a model class that is superior on an easy test is still superior on the harder external benchmark. No method achieves statistical separation at the CD = 5.538 threshold, and effect sizes are small (Cohen’s d = 0.32/0.24 for SPXY vs k-means-shuffle; Additional file 1: Table S3), so these directional results should be treated as indicative. At Sub-150, selection advantage turns negative for all splitters, indicating that at ≈120 training compounds internal test evaluation does not reliably identify the externally best model regardless of splitting strategy.

### Sub-150 sensitivity analysis

**Fig. 11:**
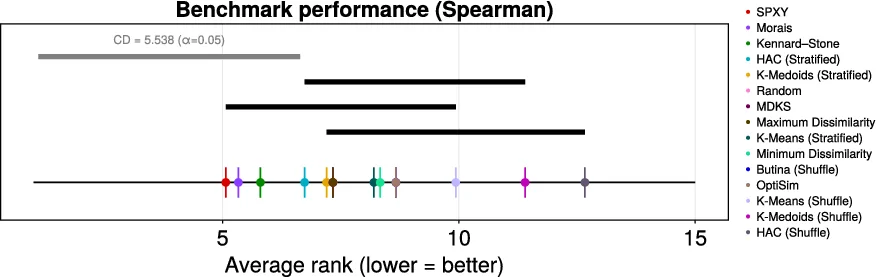
Nemenyi CD diagram for Spearman benchmark performance, Sub-150 regime (CD = 5.538, α = 0.05; lower rank = better)

**Fig. 12:**
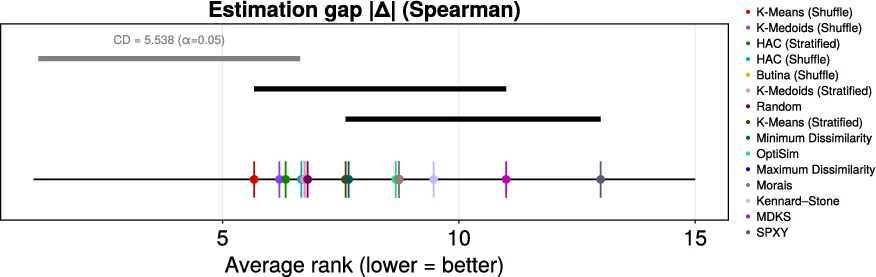
Nemenyi CD diagram for Spearman estimation error |$\Delta \rho$|, Sub-150 regime (CD = 5.538, α = 0.05; lower rank = better calibrated)

**Fig. 13:**
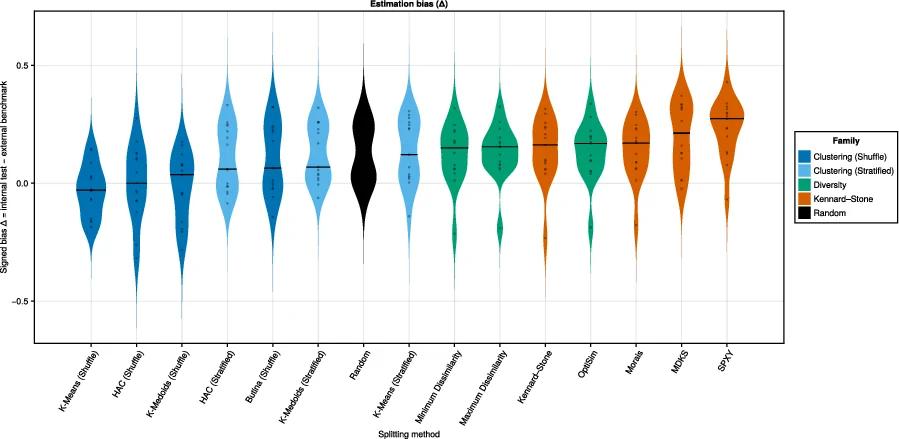
Signed Spearman estimation bias Δ = ρ_internal_test – ρ_benchmark per splitter, Sub-150 regime, sorted from best-calibrated (left) to worst (right). Dashed line: Δ = 0 (perfect calibration)

The broad family-level pattern is preserved at Sub-150 (Figs. 11, 12): KS-type and diversity methods retain the highest estimation bias (KS-type $\Delta \rho$ = +0.16-−0.27), cluster-shuffle the lowest, though individual method ranks within families shift more than at full scale. At this scale k-means-shuffle has a median $\Delta \rho$ = -−0.03, marginally pessimistic. Benchmark performance is compressed into a narrow band (range ≈ 0.05 vs ≈ 0.09 at full scale) while bias spread remains large (≈0.30), giving a bias-to-performance ratio of approximately 6×, up from 2.5× at full scale, reflecting the dominance of noise at only 150 compounds (Fig. 13). Model-selection results at Sub-150 show all methods clustered in a narrow rank band with no statistical separation whatsoever, further supporting the conclusion that splitting strategy does not affect which model class is selected (Additional file 1: Figs. S13–S14). Sub-1000 results are in agreement (Additional file 1: Figs. S15–S17).

## Discussion

### Relation to prior literature

The direction of KS-type overoptimism is qualitatively established in spectroscopic and QSAR settings [5–10]. This work adds four elements. First, bias magnitude is shown to far exceed benchmark-performance differences across a drug-discovery regression benchmark, confirming that evaluation credibility is a more sensitive function of splitter choice than realized performance. Second, the effect is general across all eleven measures spanning error, explained variance, rank correlation, and enrichment, and cannot be dismissed as an artefact of a single scoring choice. Third, the MDAS analysis (Fig. 8) provides a geometric mechanism grounding earlier qualitative arguments. Fourth, model-selection quality emerges as a distinct and opposing dimension: the worst-calibrated splitter (SPXY) numerically leads on selection advantage and hit rate, while the best-calibrated (k-means-shuffle) ranks last, though no method achieves statistical separation.

The dataset-size dependence documented in [13] is visible in our per-dataset spread (Additional file 1: Fig. S6), and the recently proposed XYOnion algorithm [25] corroborates our cluster-stratified results directionally.

### Why KS-type methods are simultaneously best on the benchmark and most optimistic

KS’s uniformly spanning training set leaves interior, structurally easy compounds as the internal test set. This inflates internal scores while making the training set maximally representative, yielding the best benchmark performance. Diversity methods have similar or lower internal MDAS to KS, keeping internal performance at a moderate level, but the external benchmark is substantially harder still, so they remain mildly optimistic; cluster-shuffle holds out whole clusters as structurally remote from training as the external benchmark, yielding near-zero bias without sacrificing representative training.

The estimation bias is largely model-agnostic: recomputing the analysis using the per-split TPE-selected model (LightGBM, random forest, KNN, SVR, or elastic net) in place of the fixed LightGBM leaves per-splitter results essentially unchanged (benchmark Spearman ρ within ≈0.01, signed bias Δ within ≈0.01; Additional file 1: Table S1). The optimism is a property of the test-set composition rather than of any particular learner.

### Implications for practice

The splitting problem divides cleanly into two tasks with different answers. The first task is estimating prospective performance. Here the evidence is statistically clear: cluster-shuffle and diversity-based methods achieve significantly better calibration across all measures. The second task is selecting the best model class when multiple classes compete: here no method achieves statistical separation and effect sizes are small (Cohen’s d ≤ 0.32; Additional file 1: Table S3), meaning the choice of splitter has no demonstrated impact on which model class is ultimately selected.

Given this framing, the practical recommendation is straightforward: cluster-shuffle splitting is the preferred default, as it achieves the best calibration while remaining competitive on benchmark performance and is not demonstrably worse for model selection; diversity-based methods are similarly well-calibrated but rank worst on benchmark performance. Practitioners who prioritize model-selection quality may use a two-stage strategy: use KS-type splitting to select the model class and hyperparameters, then re-split with cluster-shuffle and retrain the selected model to obtain a calibrated estimate of prospective performance. The relative magnitude of estimation bias versus realized-performance differences will vary with dataset size and split ratio [5, 13], and this disparity grows as datasets shrink, so the case for calibrated splitting is strongest in small-data regimes.

### Limitations

Dataset selection and benchmark: We analyze the publicly released Merck datasets, and we do not claim that the patterns described will necessarily hold for datasets outside drug-discovery regression. The Merck temporal split reflects compound testing order in a specific industrial organization and may not transfer to academic datasets or other prospective scenarios [26].

Molecular structures unavailable: Because structures are not provided, we cannot evaluate scaffold-aware or fingerprint-based splits in their native form, and whether results extend to explicit scaffold splits [27] remains an open question. More broadly, all methods here use a shared descriptor rather than the representations they would typically be deployed with; within-family rankings may therefore be descriptor-conditional.

Temporal benchmark: We treat the Merck temporal split as a proxy for prospective performance. This is a strong assumption.

Fixed model in the splitting-assessment experiment: The model-selection experiment confirms that the benchmark-performance and estimation-bias pattern is consistent across five algorithm classes under TPE optimization; whether it extends to model families not covered here remains to be verified.

Small-dataset generalizability: The Sub-150 sensitivity analysis is conducted across all 15 datasets uniformly capped at 150 development compounds; it is therefore not a direct observation of the full-data regime for any individual dataset but a controlled comparison at a fixed small-data size representative of real ADMET benchmarks. Whether findings extend to other small-data domains (e.g., non-drug-discovery regression) remains to be verified.

Fixed cluster count: All clustering-based methods use k = 10 clusters regardless of dataset size. For the largest datasets this is very coarse, making cluster-shuffle assignments closer to random. Results for the clustering family may therefore differ with other choices of k, and k-sensitivity is an open empirical question.

Nemenyi conservatism: The Nemenyi procedure is conservative; subtle real differences may not reach significance at α = 0.05. Violin plots and spread charts are provided alongside CD diagrams to convey effect sizes independently of statistical thresholds.

### Future work

Extensions that would strengthen generality include: (1) evaluation on non-drug-discovery datasets and datasets outside the Merck benchmark; (2) incorporation of structure-annotated datasets to include scaffold and pharmacophore splits; (3) validation with explicit temporal metadata within the development pool to enable comparison with temporal CV [28]; and (4) analysis of additional model classes.

## Conclusions

Across fifteen drug-discovery datasets and eleven evaluation measures, train–test splitting strategy primarily affects the reliability of performance evaluation rather than realized model generalization. Kennard–Stone-type methods achieve modest benchmark-performance gains at the cost of substantially optimistic internal estimates, confirming and quantifying the overoptimism documented in prior literature, and confirmed here as general across error, explained-variance, rank-correlation, and early-enrichment scores.

The splitting problem is best understood as two distinct tasks. For performance estimation, the evidence is statistically clear: cluster-shuffle methods produce significantly better-calibrated estimates. For model selection among competing model classes, no splitting method achieves statistical separation and effect sizes are small, meaning the choice of splitter has no demonstrated impact on which model class is ultimately selected. Cluster-shuffle is therefore the recommended default: it wins decisively on calibration and is not demonstrably worse for model selection. Practitioners who wish to optimize model selection explicitly may use a two-stage strategy: KS-type splitting for model and hyperparameter selection, followed by re-splitting with cluster-shuffle and retraining to obtain a calibrated prospective performance estimate.

## Supplementary Information

Supplementary material 1.

## Data Availability

The molecular activity datasets analysed in this study are the publicly released portion of the Merck molecular activity benchmark and are available from the original publication (DOI: 10.1021/ci500747n). The analysis code, splitting implementations, and figure-generation scripts are available at https://github.com/davide-grheco/split-performance-evaluation. Experimental results and pre-computed splits are deposited on Zenodo (doi:10.5281/zenodo.20705244).

## Abbreviations

AD: Applicability domain
BEDROC: Boltzmann-enhanced discrimination of receiver operating characteristic
CD: Critical difference
CV: Cross-validation
EF: Enrichment factor
HAC: Hierarchical agglomerative clustering
KS: Kennard–Stone
MAE: Mean absolute error
MDAS: Mahalanobis Distribution Alignment Score
MDKS: Modified Kennard–Stone
MedAE: Median absolute error
NN: Nearest neighbor
QSAR: Quantitative structure–activity relationship
QSPR: Quantitative structure–property relationship
RBF: Radial basis function
RMSE: Root-mean-square error
SPXY: Sample set partitioning based on joint x–y distances
SVR: Support vector regressor
TPE: Tree-structured Parzen estimator
