# Supplementary material for: Train–test splits matter more for evaluation than for performance
Source: J Cheminform. 2026 Aug 4;18:152. doi: 10.1186/s13321-026-01266-7 (PMC13726078; doi:10.1186/s13321-026-01266-7)
Supplement: Supplementary file 1 [file 13321_2026_1266_MOESM1_ESM.pdf]

Additional file 1: Supplementary Information

Train–Test Splits Matter More for Evaluation Than for Performance

This file contains supplementary figures and tables supporting the main manuscript. Figures S1–S12 accompany the main full-data analysis. Figures S13–S14 provide additional model-selection results for the Sub-150 sensitivity analysis. Figures S15–S17 provide the Sub-1000 sensitivity analysis. Figures S18–S33 show Nemenyi CD diagrams for all eight remaining measures not featured as the primary worked example in the main text, confirming the three-tier family ordering reported for Spearman ρ. Tables S1–S3 provide numerical summaries of the main experiments, and Tables S4–S5 give the full multicriteria decision-analysis rankings.

Benchmark Performance

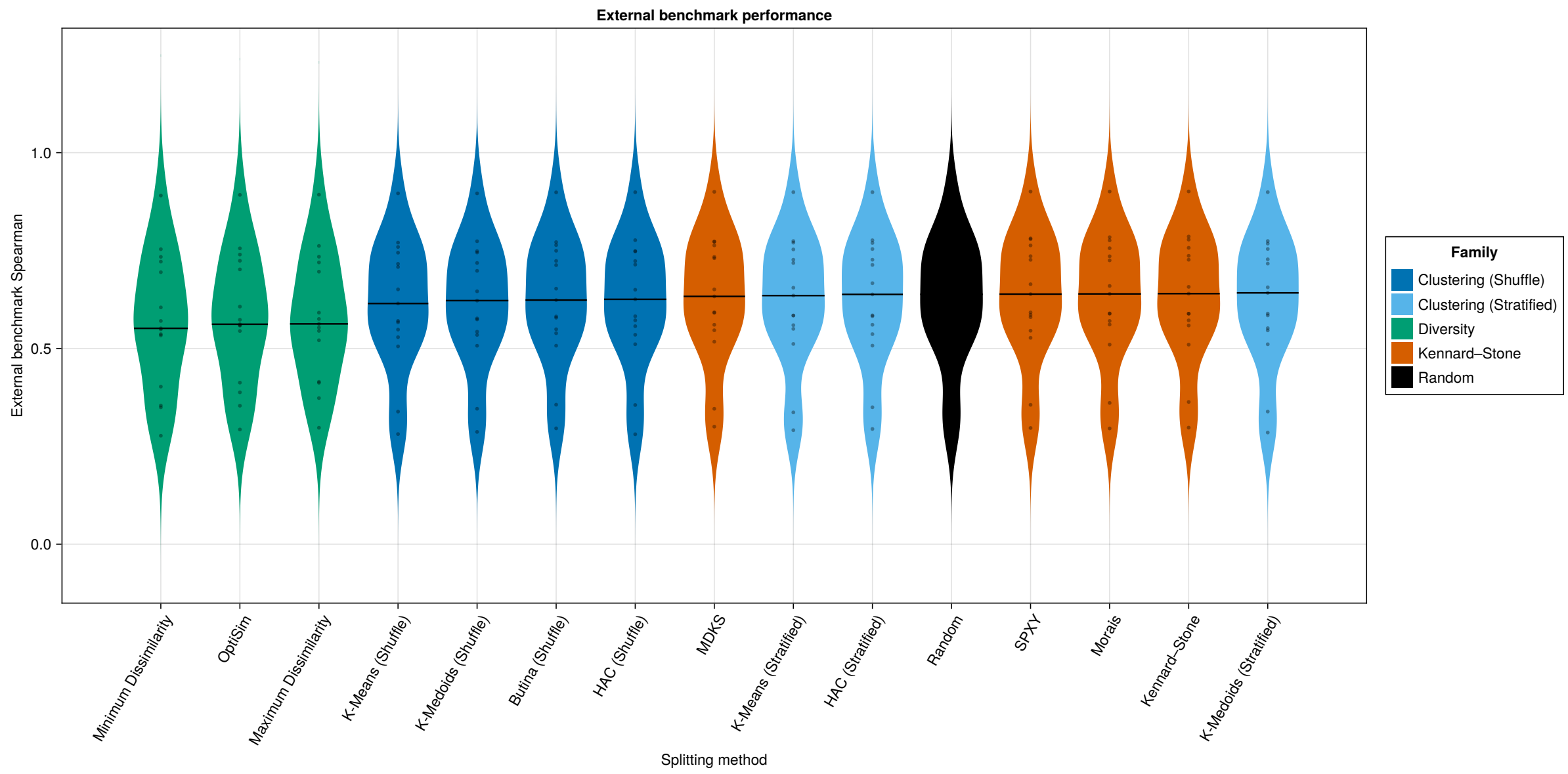

Figure 1: Distribution of benchmark Spearman ρ across all (dataset, CV base set) combinations, one violin per splitter, ordered by mean performance rank (best at right). The rightmost violins (KS, SPXY, MDKS, Morais) are shifted slightly upward relative to diversity methods (leftmost). Medians differ by approximately $\Delta\rho \approx 0.09$ between best and worst splitter; within-method variance across datasets substantially exceeds between-method median differences, indicating that dataset identity dominates realized benchmark performance.

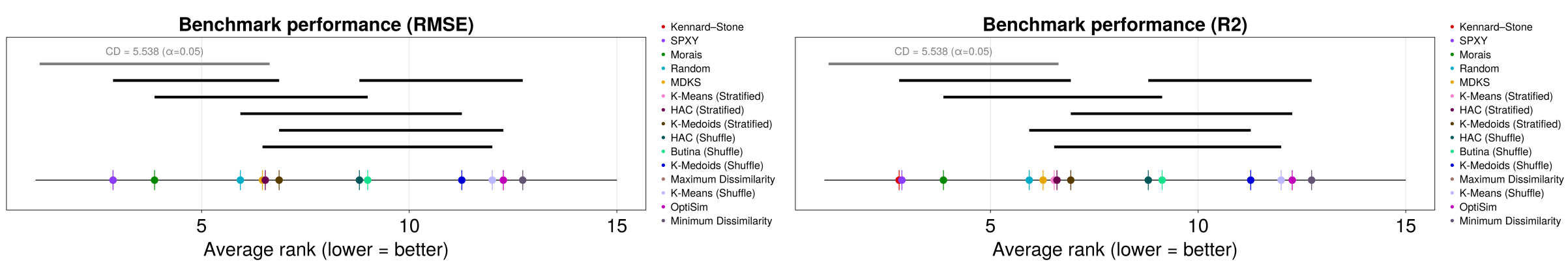

Figure 2: CD diagrams: external benchmark RMSE (left) and R^2 (right). Left: lower rank = lower (better) RMSE. Right: lower rank = higher (better) R^2. Both orderings mirror the Spearman benchmark CD diagram: KS-type methods at ranks 1–5 (RMSE) and ranks 1–4 (R^2), diversity-based methods at ranks 12–15, cluster-shuffle intermediate. There is no measure-dependent reversal.

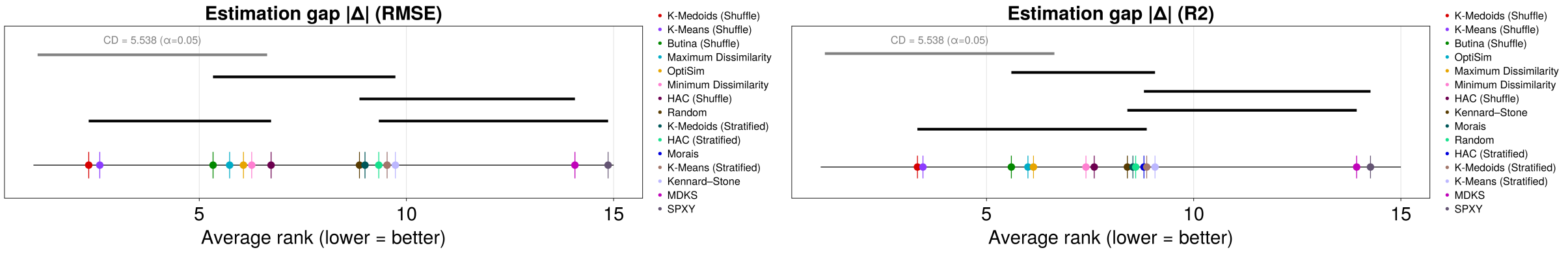

Figure 3: CD diagrams: RMSE estimation error |ΔRMSE| (left) and R^2 estimation error $|\Delta R^2|$ (right). Left: lower rank = smaller |ΔRMSE| = better calibration; cluster-shuffle and diversity at ranks 1–8 (best calibrated); KS-type at ranks 12–15 (worst). Right: k-means- and k-medoids-shuffle lead (ranks $\approx$ 3.3–3.5); SPXY and MDKS (ranks $\approx$ 14) are significantly worse. All methods show optimistic R^2 estimates, with KS-type the most optimistic ($\Delta R^2 \approx$ +0.26–+0.41) and k-means/k-medoids-shuffle near-zero ($\Delta R^2 \approx$ +0.03).

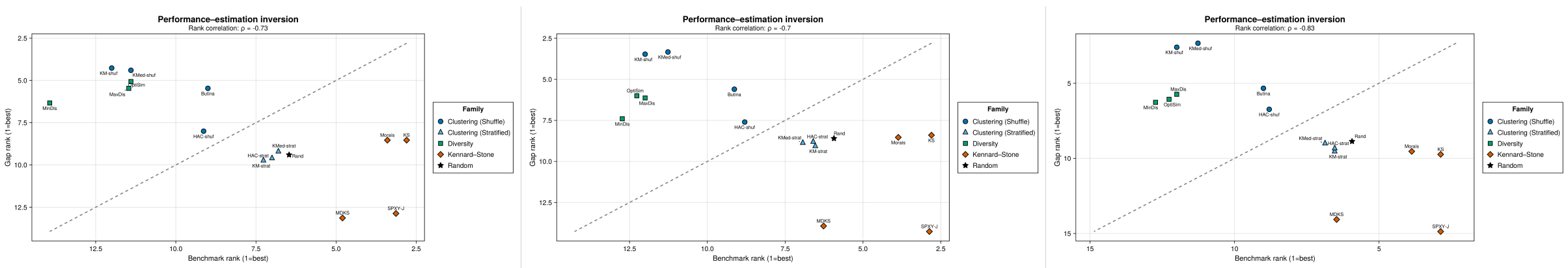

Figure 4: Performance–estimation inversion for Spearman ρ (left), R^2 (center), and RMSE (right). Each point = one splitter; x = mean rank on benchmark performance (lower = better); y = mean rank on estimation error (lower = better). Rank correlations: Spearman $\rho = -0.73$, R^2 $\rho = -0.70$, RMSE $\rho = -0.83$. KS-type methods consistently cluster at bottom-right (good benchmark rank, worst calibration); k-means/k-medoids-shuffle at top-left (near-best benchmark, best calibration). The inversion holds across all measures.

Performance-Estimation Bias

Per-Dataset Analysis

Distributional Distance Analysis

Applicability Domain

All-Measures Summary

Geometric Basis of Calibration

Nemenyi Pairwise Tests

Sub-150 Sensitivity Analysis: Model Selection

The two figures below supplement the Sub-150 sensitivity analysis reported in the main text. At $\approx$120 training compounds, internal test evaluation does not reliably identify the externally best model regardless of splitting strategy: selection advantage turns negative for all splitters, all methods cluster in a narrow rank band on both the model-selection CD diagram and the champion-selection chart, and no pair is statistically separated at the CD = 5.538 threshold.

Sub-1000 Sensitivity Analysis

The three figures below correspond to the Sub-1000 sensitivity analysis described in the Methods (capping every outer CV base set at 1,000 compounds). This analysis is provided as a secondary size-robustness check; the primary sensitivity analysis at 150 compounds (Sub-150), which better represents the scale of real-world ADMET and early-stage drug-discovery datasets, is reported in the main text. The family-level ordering is the same at 1,000 compounds as at full scale: KS-type methods remain best on benchmark and worst-calibrated; k-means- and k-medoids-shuffle remain

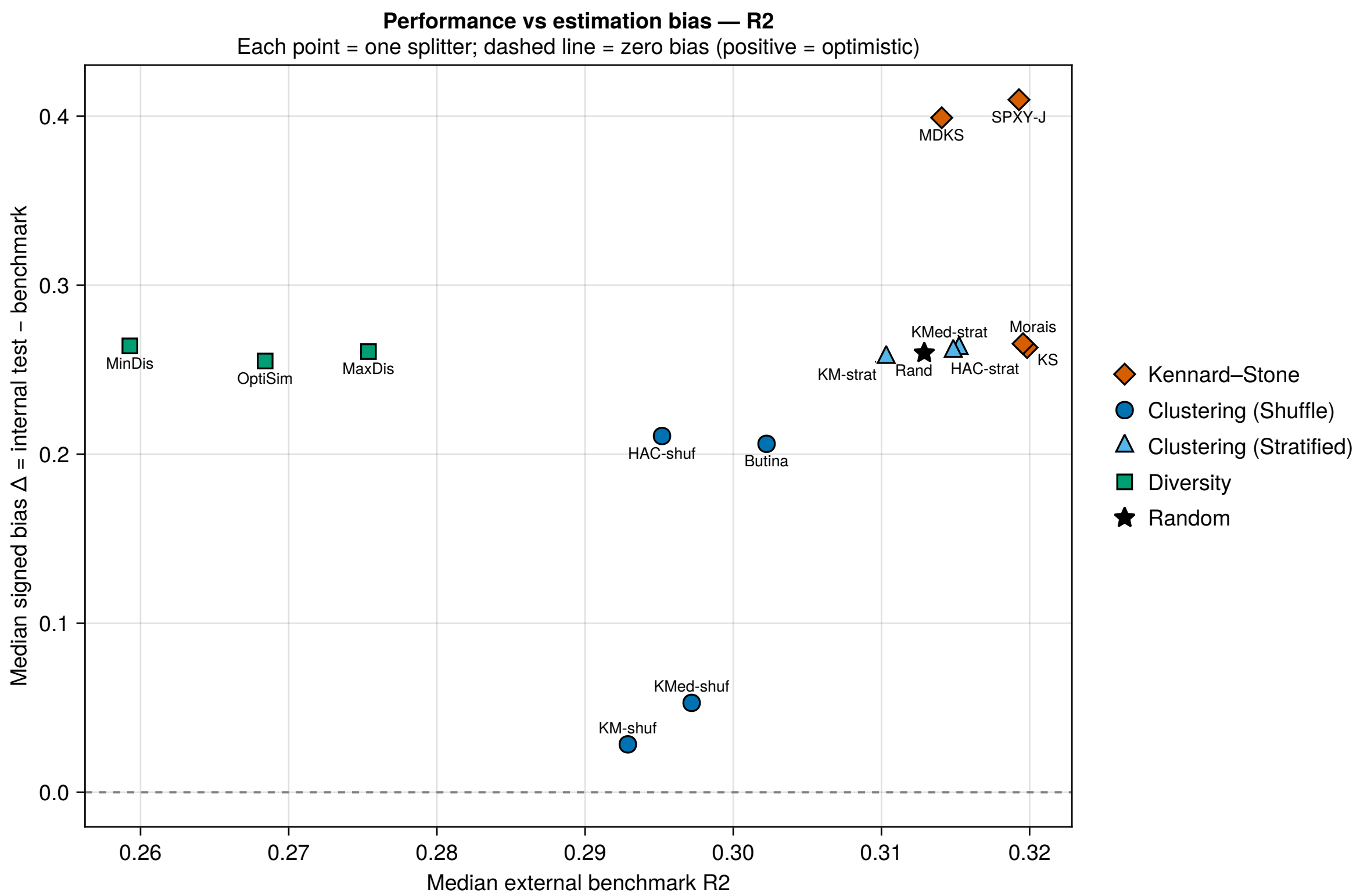

Figure 5: Benchmark performance vs signed estimation bias (R^2). Each point = one splitter; x = median benchmark R^2; y = median signed bias ΔR^2 = internal test − benchmark. Dashed line at $\Delta R^2 = 0$. KS-type methods cluster at high R^2 with large positive bias ($\Delta R^2 \approx +0.26$–$+0.41$); k-means/k-medoids-shuffle sit near $\Delta R^2 \approx +0.03$. The performance–calibration tradeoff pattern is identical to the Spearman case.

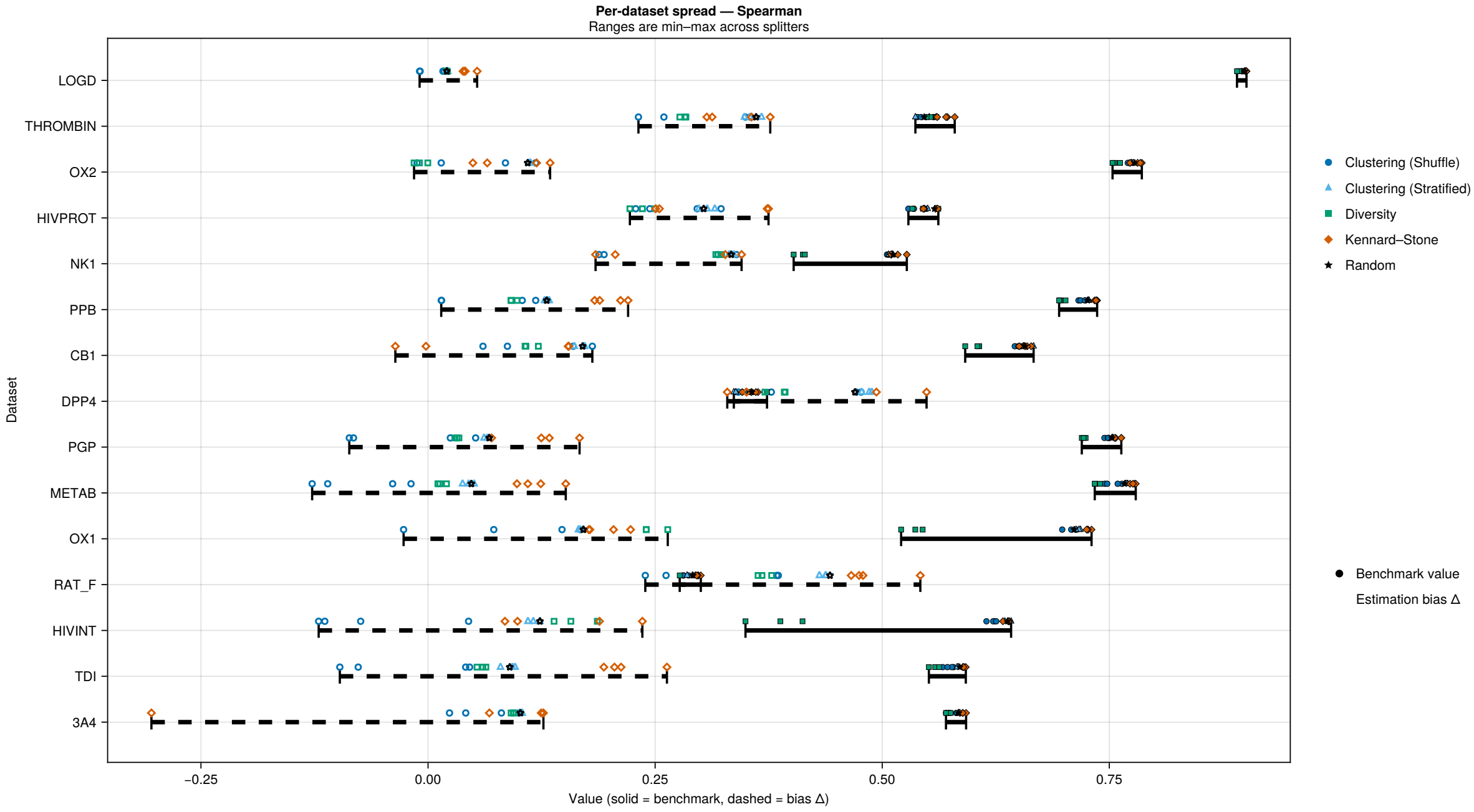

Figure 6: Per-dataset spread: Spearman ρ. Horizontal ranges show the min–max spread of benchmark performance (solid line) and estimation bias Δ (dashed line) across all splitters within each dataset. The qualitative pattern (KS-type at right = high benchmark ρ; diversity at left = low benchmark ρ) is preserved across all 15 datasets. HIVINT and METAB (smallest datasets) show the widest relative spread in both benchmark performance and estimation bias, consistent with the known size-dependence of split effects. The large datasets (3A4, LOGD) show compressed spread in benchmark performance but retain the directional ordering. This within-dataset variability motivates the across-dataset Nemenyi analysis as the primary inferential tool.

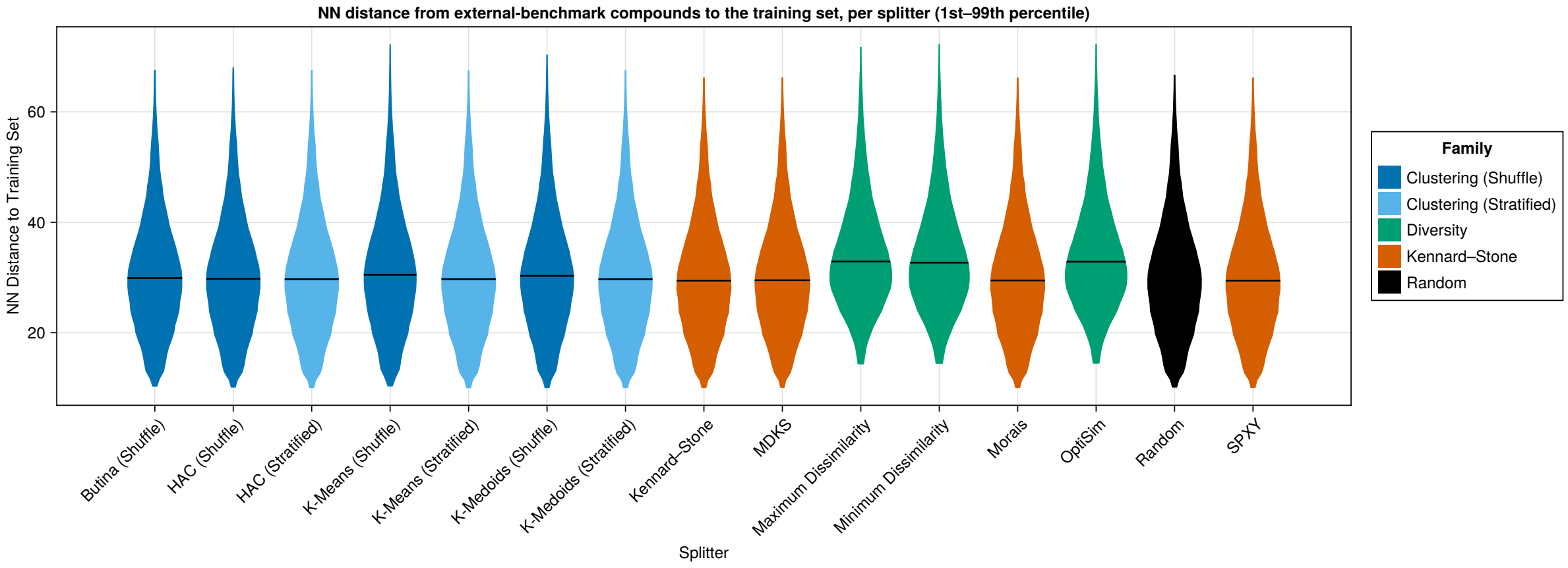

Figure 7: NN distance violin plots: nearest-neighbor Euclidean distance from external benchmark compounds to the training set, by splitting method. Diversity-based methods produce the highest NN distances (benchmark compounds structurally remote from training); cluster-shuffle methods are intermediate; KS-type methods produce the lowest NN distances (benchmark compounds closest to the training manifold). This confirms the test-set composition picture quantified by the Mahalanobis analysis.

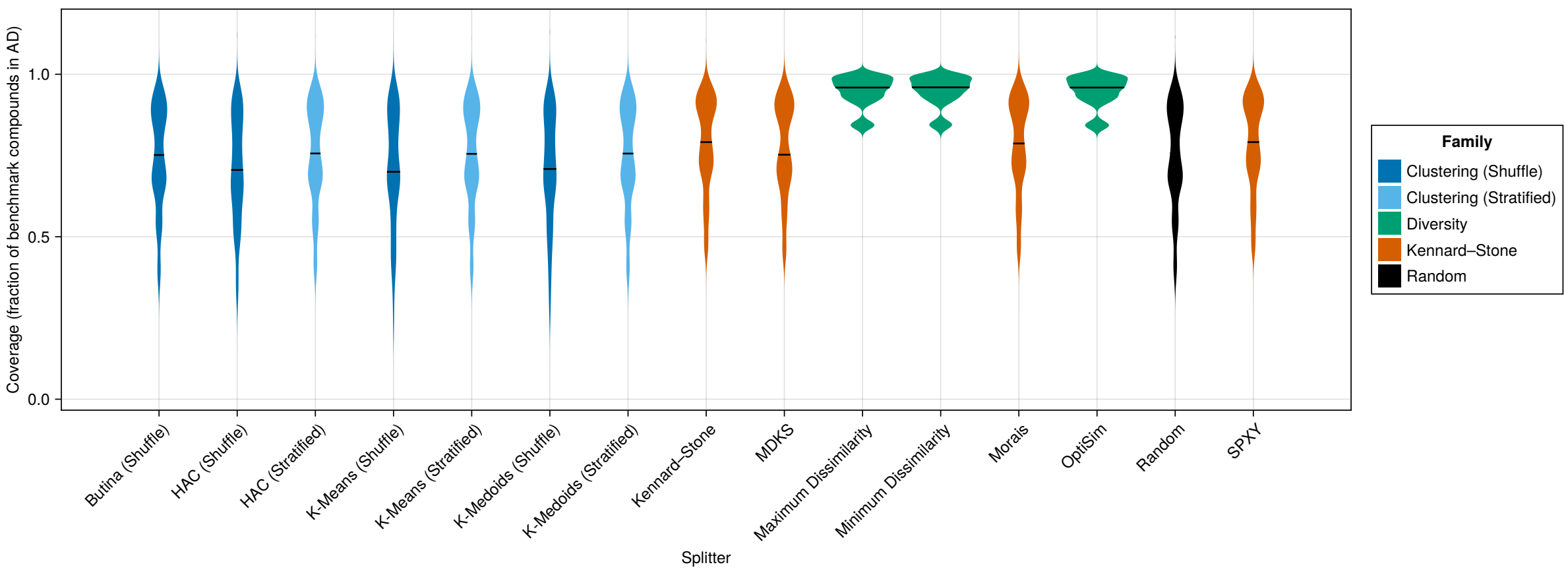

Figure 8: AD coverage by splitting method: fraction of external benchmark compounds within the model's k-NN applicability domain. Diversity-based methods achieve the highest median coverage (broad training manifold); KS-type methods are intermediate; cluster-shuffle methods the lowest.

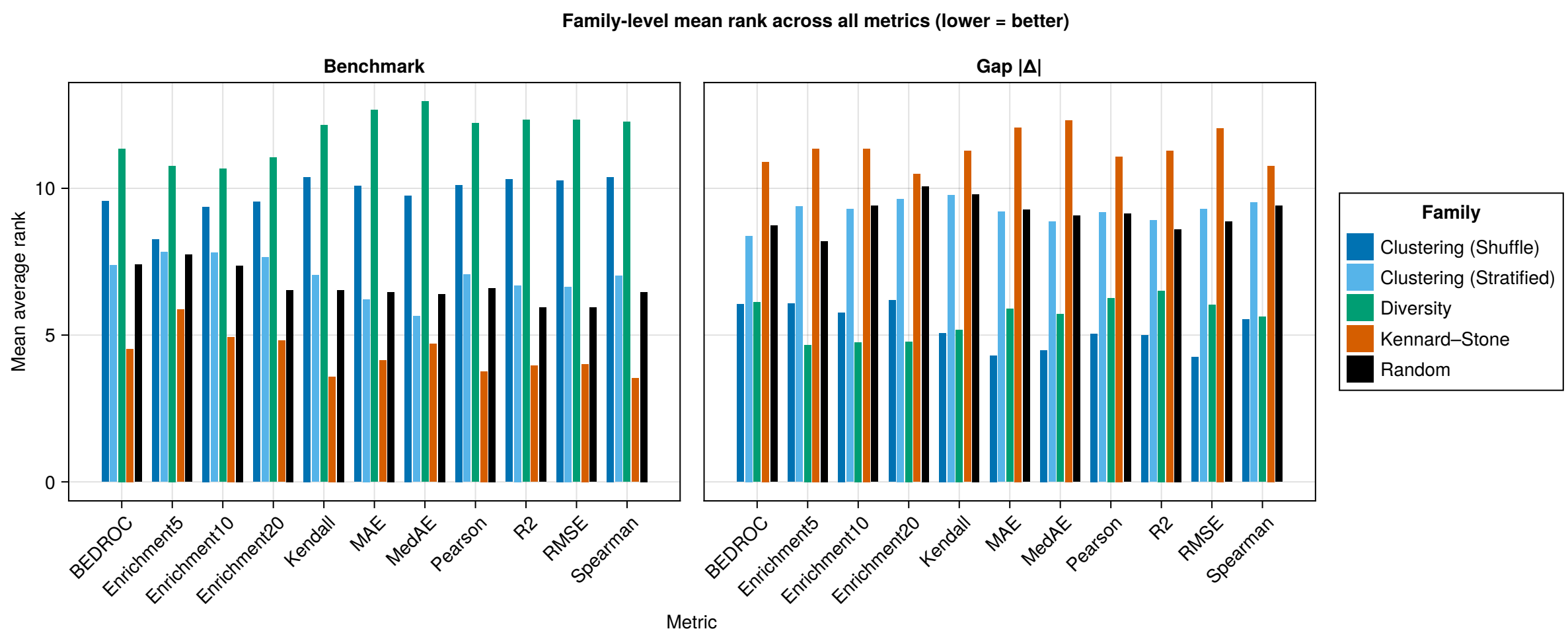

Figure 9: Family-level mean rank bar chart across all measures and all splitting strategies. Cluster-shuffle is the only family that achieves competitive mean benchmark rank and best calibration rank simultaneously across all measures. KS-type methods win on benchmark across every measure but have the worst gap rank; diversity-based methods have the worst benchmark rank but a competitive gap rank. Measures: RMSE, R^2, MAE, MedAE, Pearson, Spearman, Kendall, Enrichment-5/10/20%, BEDROC.

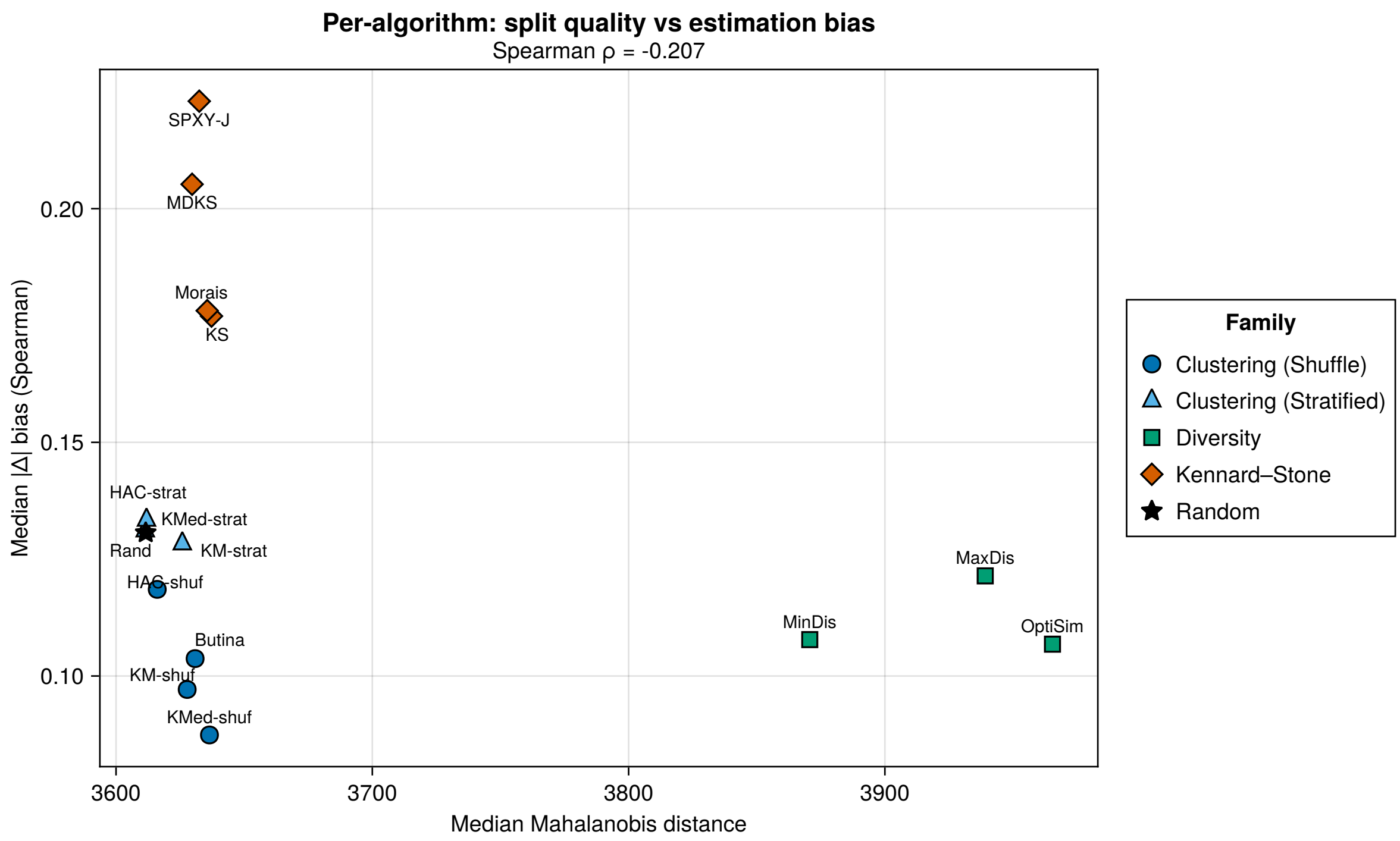

Figure 10: Per-algorithm Mahalanobis distance vs estimation bias: each point = one splitter. Methods whose external MDAS is close to their internal MDAS ($x \approx y$) show smaller $|\Delta \text{Spearman}|$. KS-type methods are separated: high external MDAS (hard external benchmark) but low internal MDAS (easy internal test set), producing large bias. Cluster-shuffle methods sit near the diagonal: internal and external MDAS are matched, explaining their near-zero bias.

best-calibrated. Absolute bias magnitudes are larger (median SPXY $\Delta\rho \approx +0.30$ vs $\approx +0.22$ at full scale), and the critical difference is unchanged, but no directional comparison reverses.

Per-Measure Nemenyi CD Diagrams

The following figures show Nemenyi CD diagrams for the eight remaining measures not featured as the primary worked example in the main text (RMSE and R^2 are shown in combined form as Figures S2–S3 above). In every case the three-tier family structure is preserved: KS-type methods lead on benchmark and are worst-calibrated; diversity-based methods are worst on benchmark and well-calibrated; cluster-shuffle methods are intermediate on benchmark and best-calibrated. This confirms that the inversion holds across all measures rather than being an artefact of any single scoring choice. All diagrams use CD = 5.538 at $\alpha = 0.05$ ($k = 15$ algorithms, $n = 15$ datasets).

Error Measures

Correlation Measures

Enrichment Measures

Supplementary Tables

Table S1. Robustness of benchmark performance and estimation bias to the choice of model. Per-splitter **mean** (over the 15 datasets) external-benchmark Spearman ρ and signed estimation bias Δ = internal − benchmark, computed with the fixed LightGBM of the splitting-assessment experiment versus the per-split TPE-selected model of the model-selection experiment (chosen among LightGBM, random forest, KNN, SVR, and elastic net). Note: the main text cites **median** bias values, which differ from the means shown here because the per-dataset bias distributions are right-skewed (a small number of datasets show substantially higher bias, pulling the mean

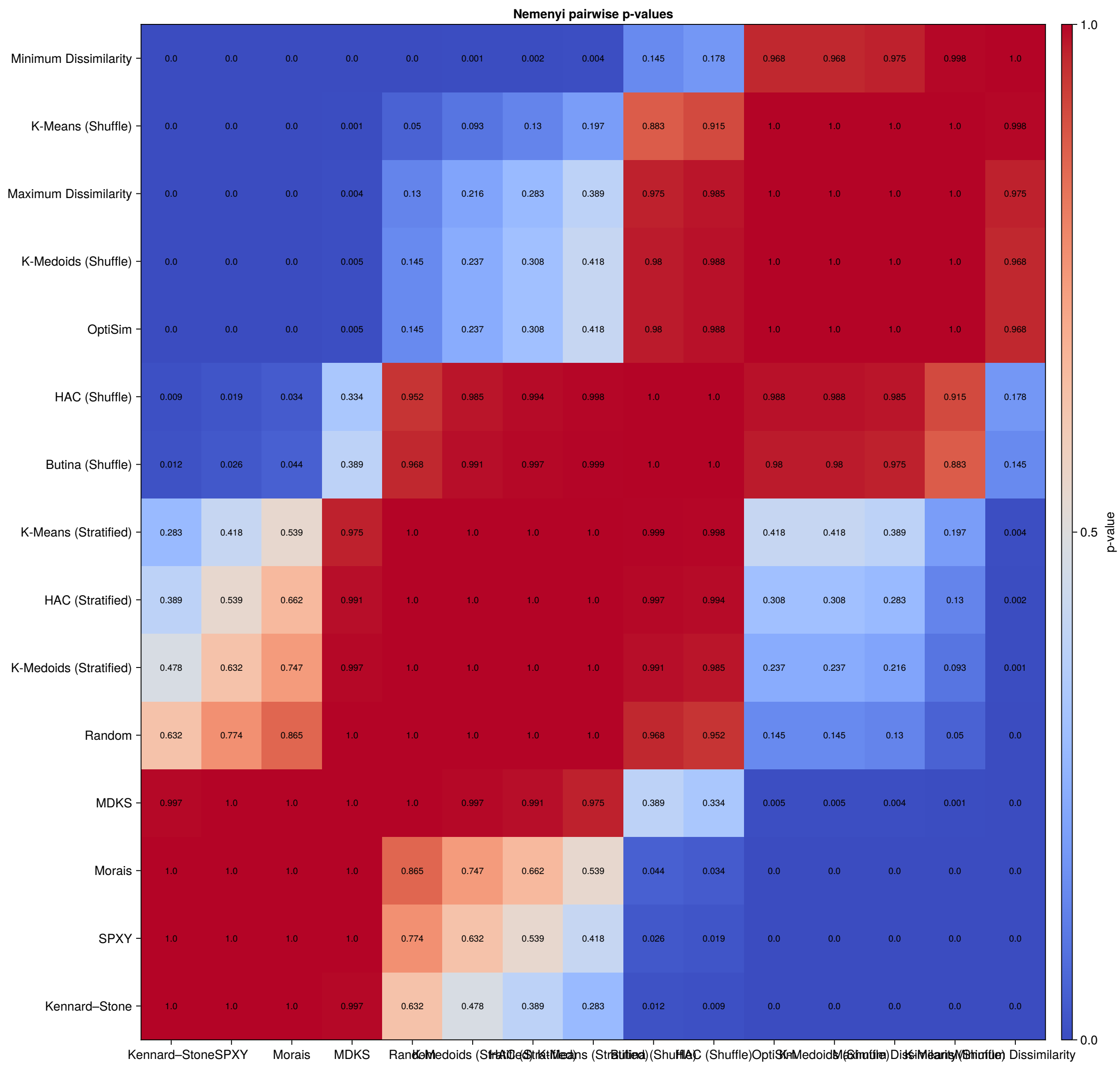

Figure 11: Nemenyi pairwise p-value matrix: Spearman benchmark performance. Green = not distinguishable (high p); red = significant difference (low p). Algorithms ordered by average rank (best top-left). The block structure confirms the CD diagram interpretation: KS-type methods (top rows) are not significantly different from each other but are significantly different from diversity-based methods (bottom rows) at α = 0.05.

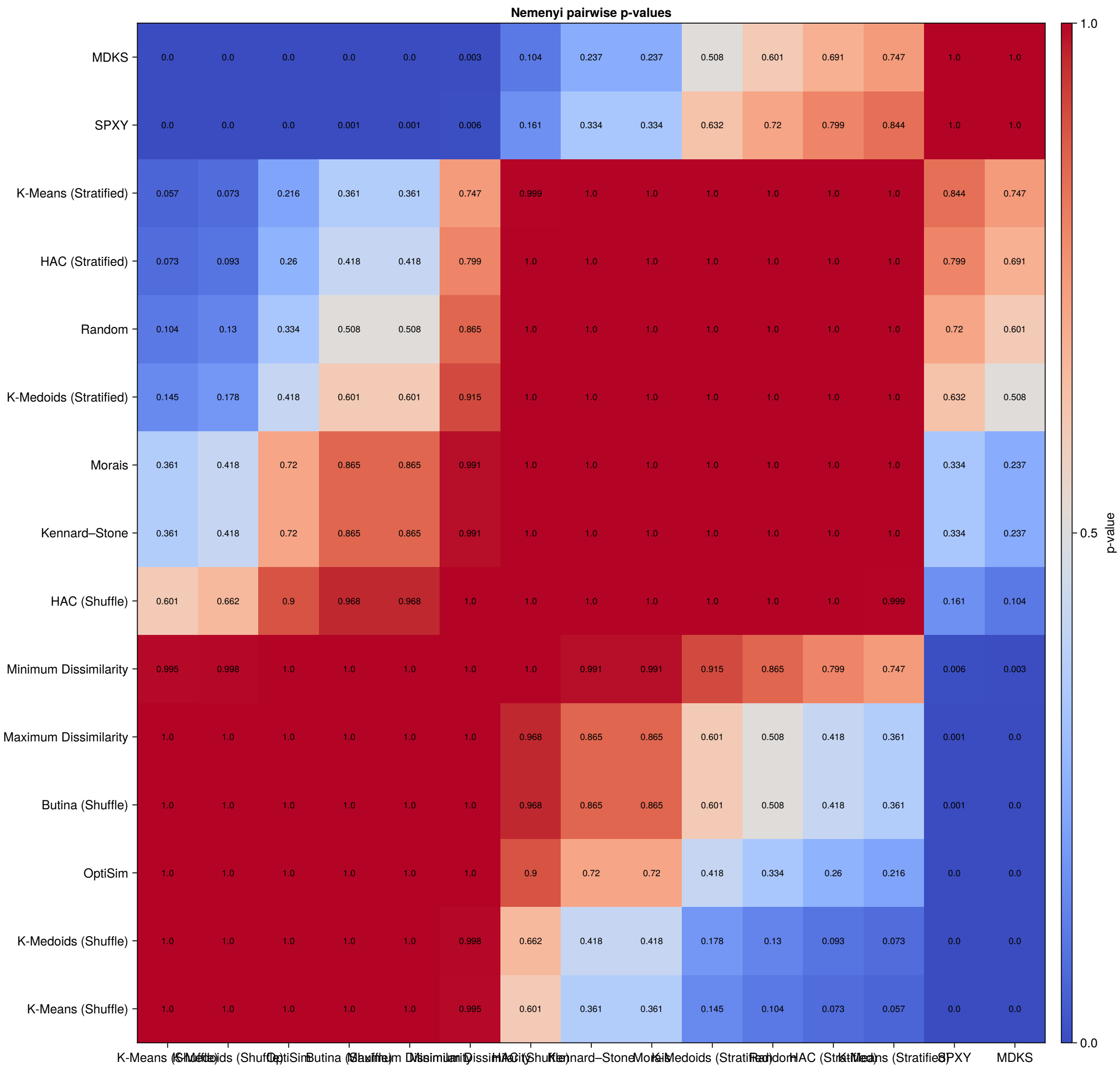

Figure 12: Nemenyi pairwise p-value matrix: Spearman estimation error |ΔSpearman|. Green = not distinguishable; red = significantly different. Algorithms ordered by average gap rank (best calibrated top-left). The matrix mirrors Fig. S11 with families exchanged: diversity-based and cluster-shuffle methods (top rows, best calibrated) are indistinguishable from each other but significantly better calibrated than SPXY and MDKS (bottom rows). SPXY is significantly worse than nearly every other method.

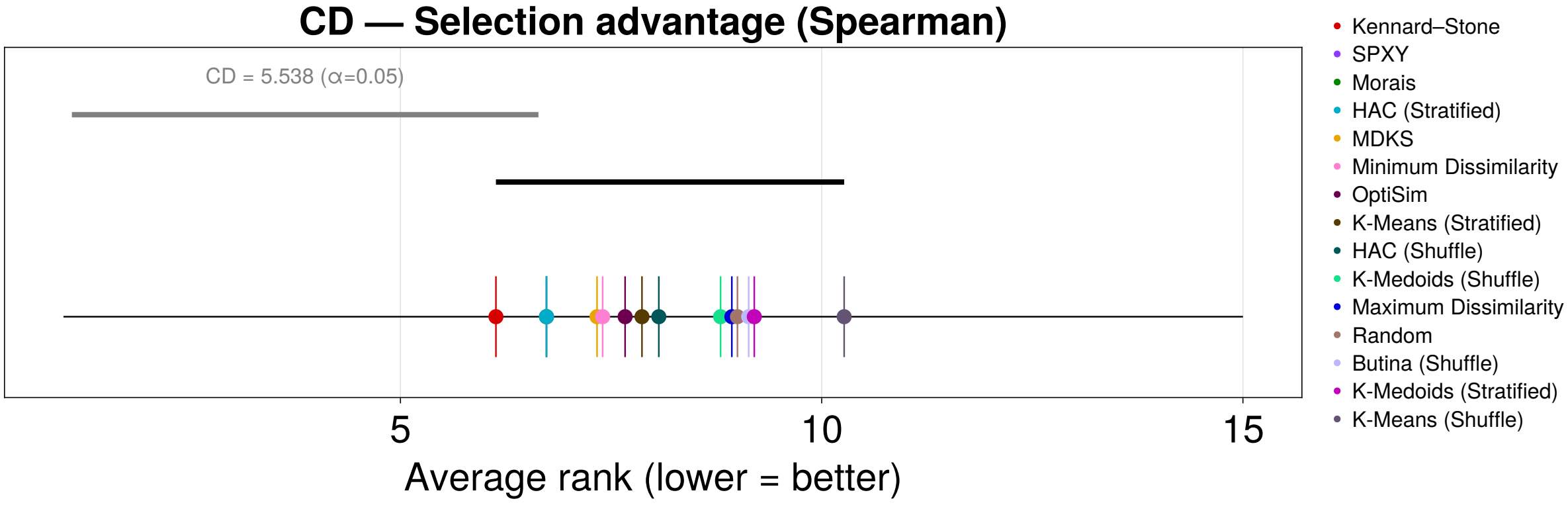

Figure 13: Nemenyi CD diagram for model-selection advantage, Sub-150 regime. All fifteen splitting strategies collapse into a single statistically indistinguishable group; no method achieves separation at the CD = 5.538 threshold. This confirms that at 150 training compounds, splitting strategy has no demonstrated impact on which model class is selected.

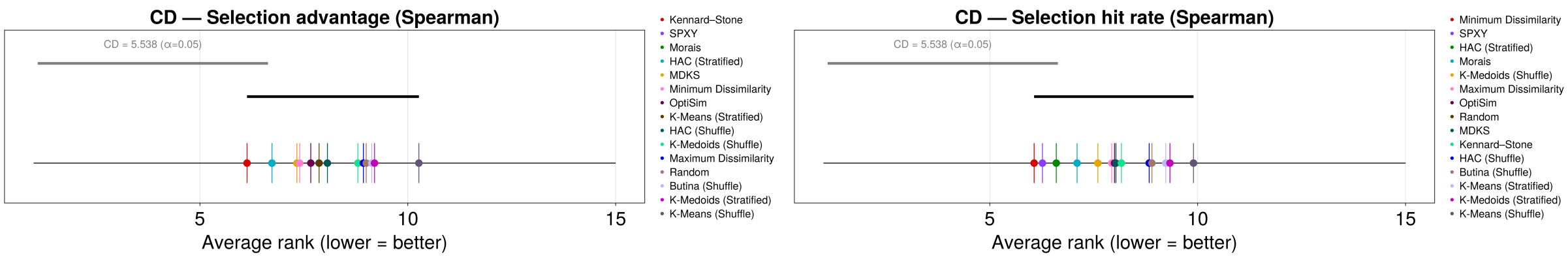

Figure 14: Model-selection quality (selection advantage and hit rate), Sub-150 regime. Left: selection advantage (external Spearman ρ of selected model minus best runner-up). Right: hit rate (fraction of folds where selected model is also externally best). All methods show negative or near-zero median selection advantage; hit rates cluster in a narrow band. The directional ordering seen at full scale (SPXY leading, k-means-shuffle last) disappears at this sample size.

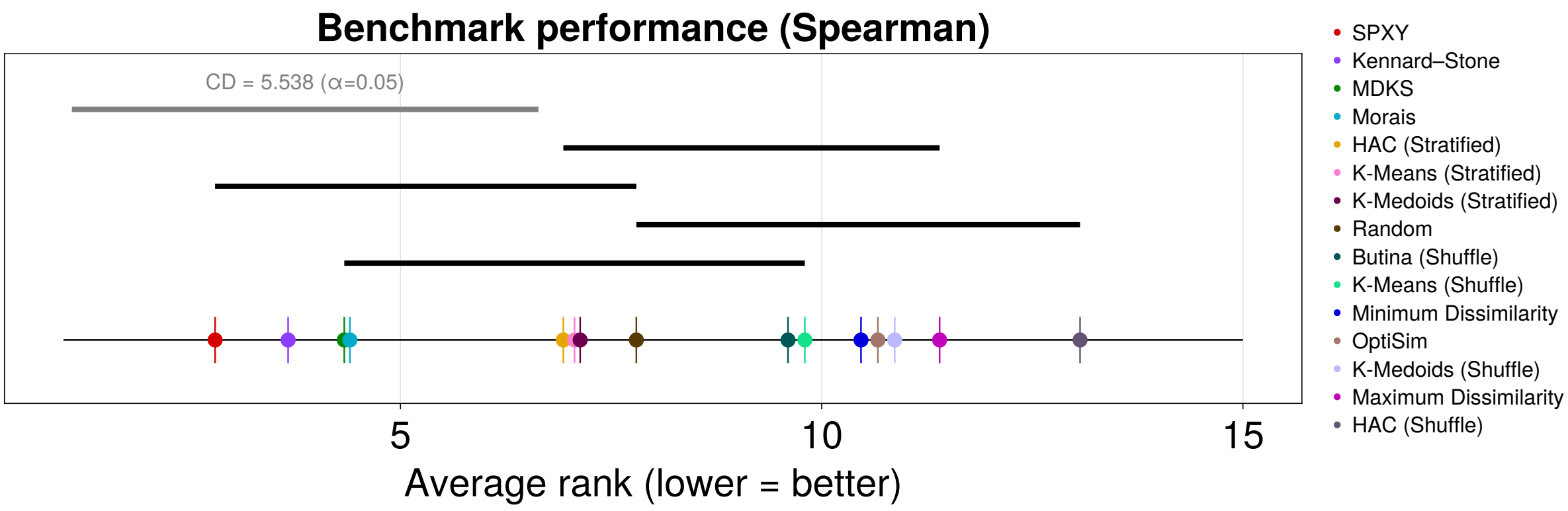

Figure 15: CD diagram: Spearman benchmark performance, Sub-1000 regime. Development sets capped at 1,000 compounds. KS-type methods again lead (KS rank ≈ 2–3, SPXY ≈ 2–3), diversity-based methods are again worst (MaxDiss/MinDiss/OptiSim ranks ≈ 12–14), and cluster-shuffle occupies the middle. The CD = 5.538 is identical to the full-data analysis (k = 15 algorithms, n = 15 datasets); no family reversal is observed.

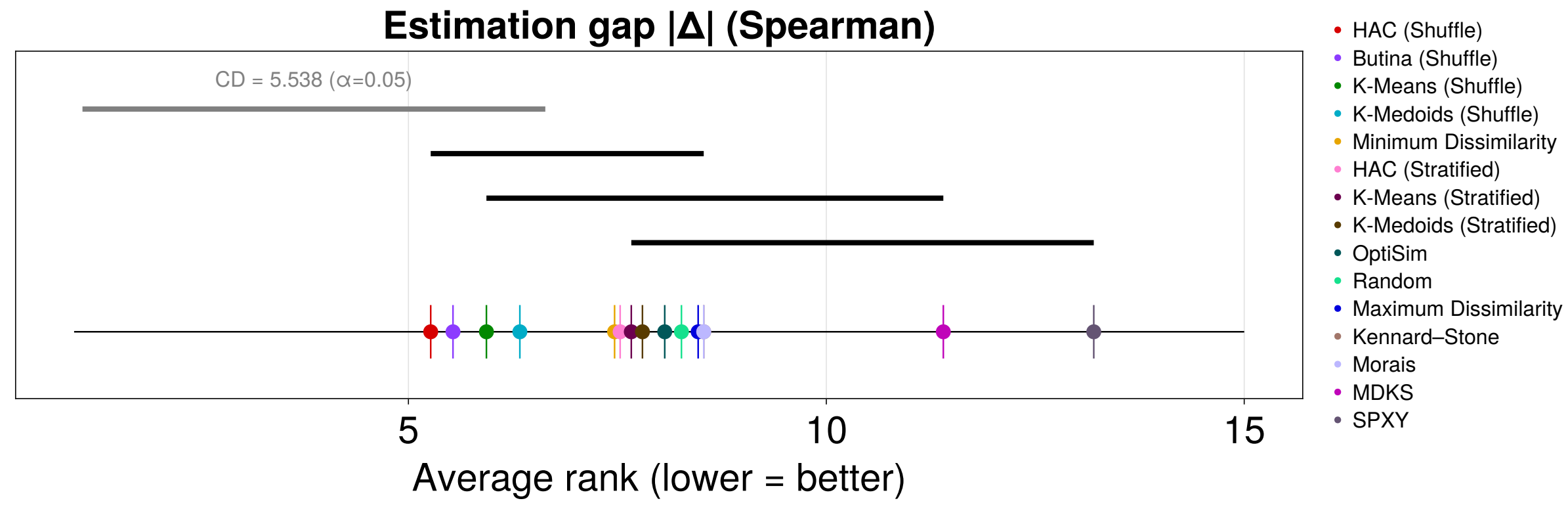

Figure 16: CD diagram: Spearman estimation error |ΔSpearman|, Sub-1000 regime. The mirror structure of the full-data analysis is preserved: diversity and cluster-shuffle methods rank best (smallest |ΔSpearman|), KS-type methods worst (largest), SPXY significantly separated from the calibrated group. Absolute estimation errors are larger under Sub-1000 conditions.

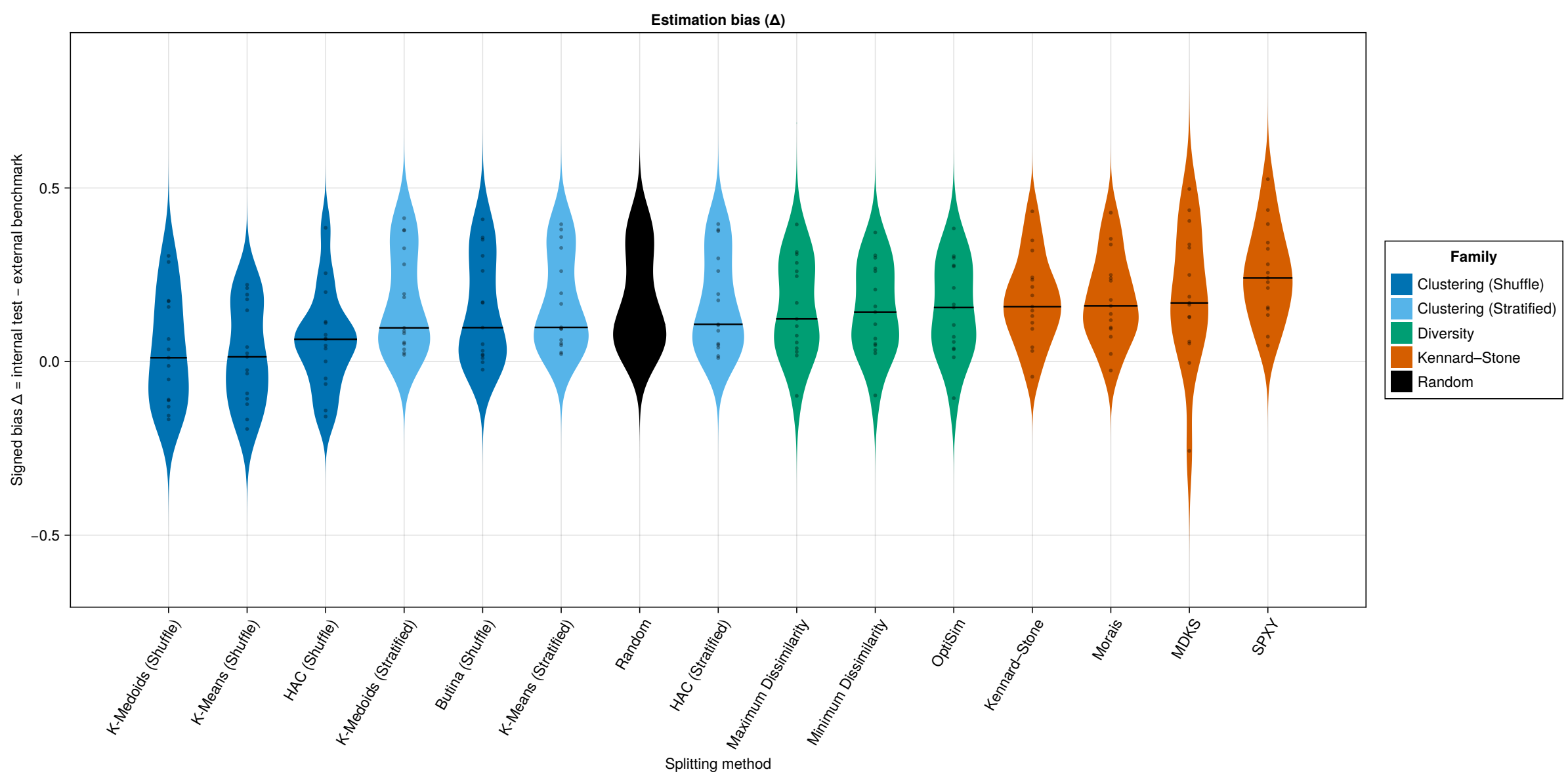

Figure 17: Signed Spearman estimation bias violin plots, Sub-1000 regime. Every method remains optimistic ($\Delta > 0$); bias magnitudes are larger than under full-data conditions. k-means- and k-medoids-shuffle again have the smallest median bias ($\Delta \approx +0.06$–0.07 vs. $\approx +0.02$ full-data), while SPXY has the largest ($\Delta \approx +0.30$). The rank order of method families is unchanged from the full-data analysis.

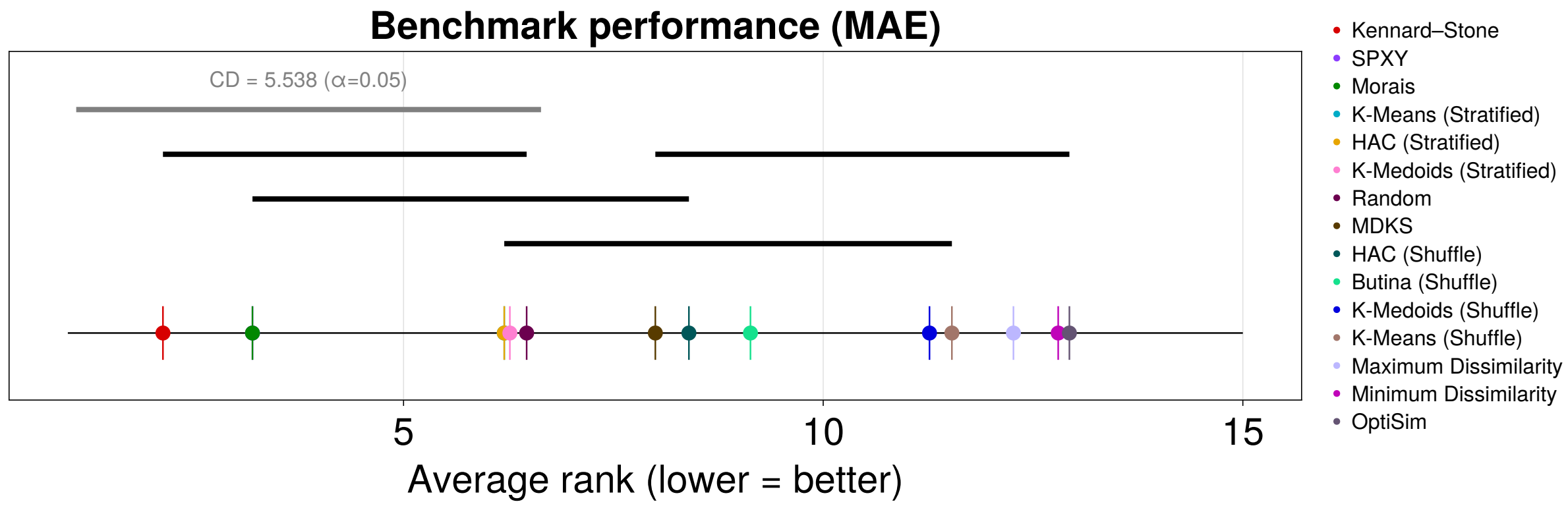

Figure 18: CD diagram: MAE benchmark performance (lower rank = lower MAE = better). Family ordering mirrors the Spearman benchmark result: KS-type methods lead (ranks 1–4), diversity-based methods are last (ranks 12–15), cluster-shuffle intermediate.

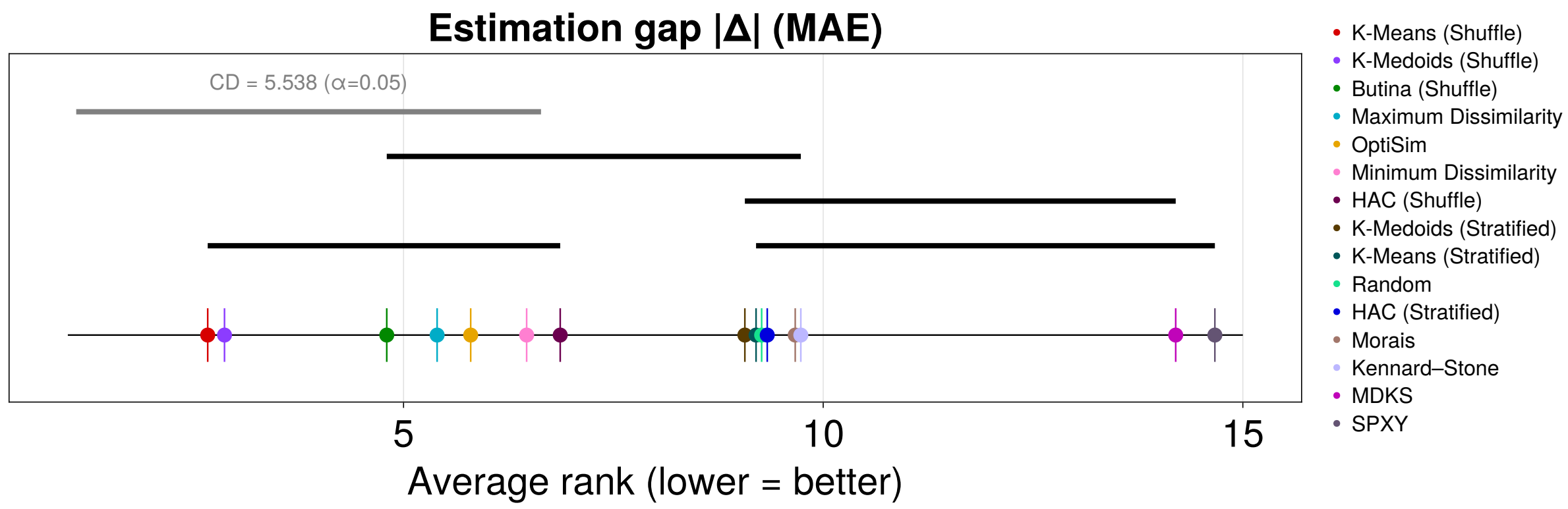

Figure 19: CD diagram: MAE estimation error $|\Delta\text{MAE}|$ (lower rank = smaller $|\Delta\text{MAE}|$ = better calibrated). Family ordering mirrors the Spearman estimation-error result: k-means- and k-medoids-shuffle lead, KS-type methods are worst-calibrated.

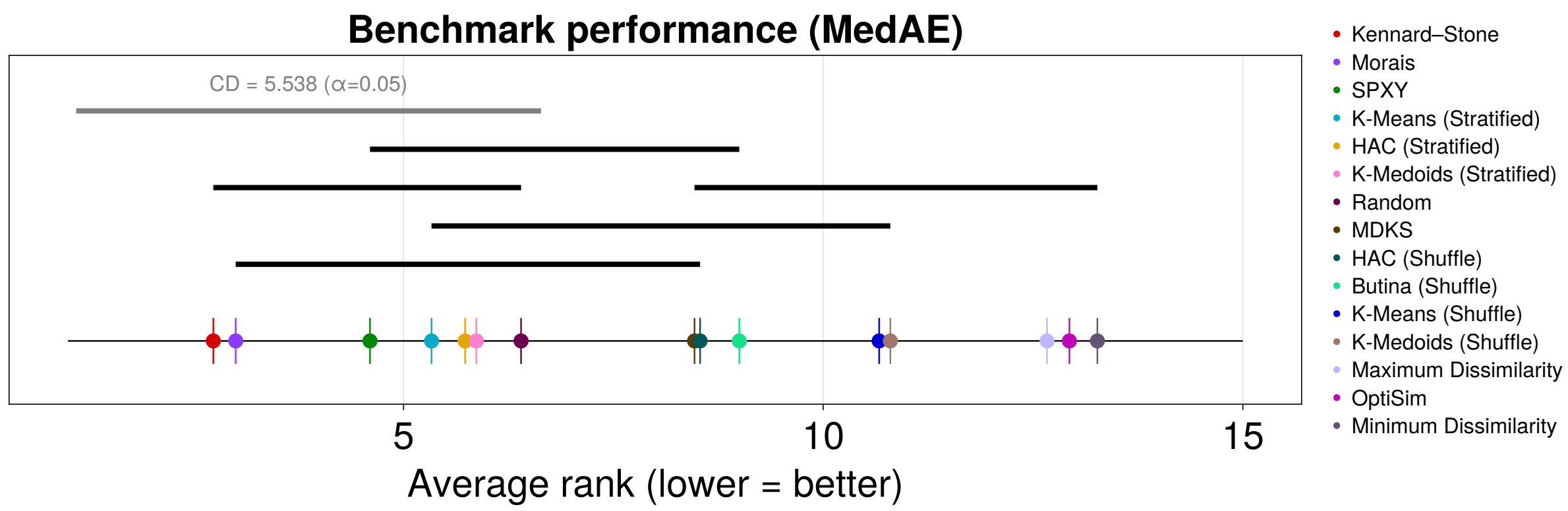

Figure 20: CD diagram: MedAE benchmark performance. Family ordering is consistent with RMSE and MAE: KS-type lead, diversity last, cluster-shuffle intermediate.

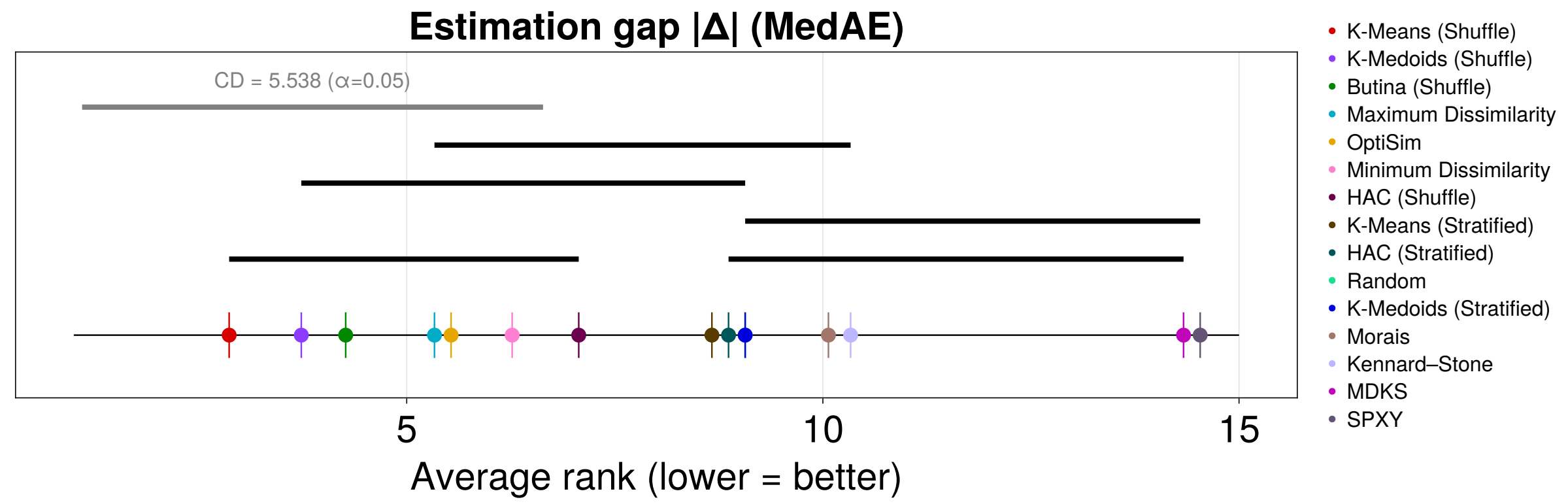

Figure 21: CD diagram: MedAE estimation error |ΔMedAE|. Calibration ordering mirrors RMSE and MAE: k-means/k-medoids-shuffle best calibrated; KS-type worst.

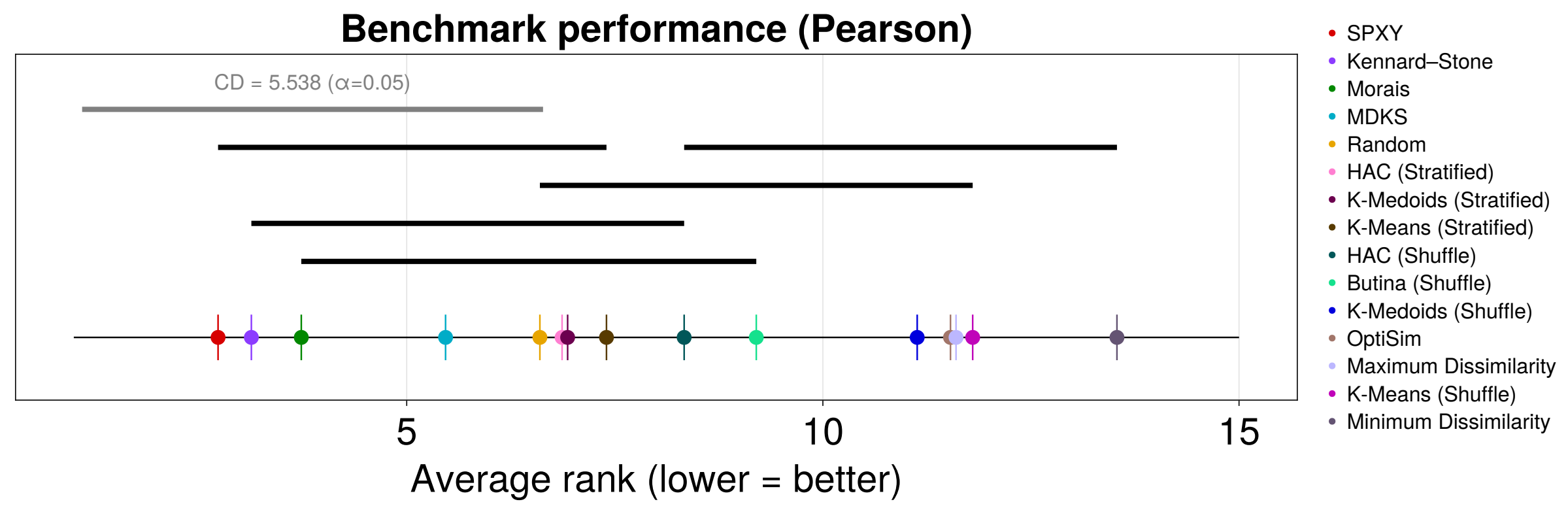

Figure 22: CD diagram: Pearson r benchmark performance (lower rank = higher r = better). Family ordering is identical to Spearman: KS-type lead, diversity last. The similarity between Spearman and Pearson orderings confirms that the family ranking is not sensitive to the choice of rank-based versus linear correlation.

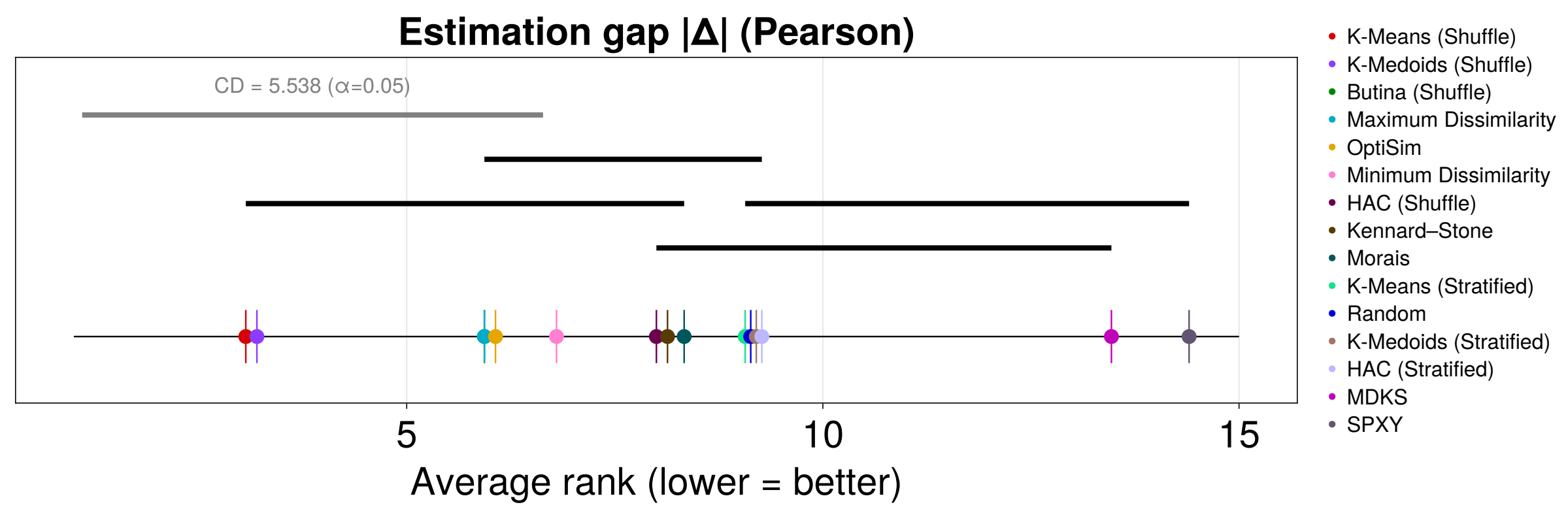

Figure 23: CD diagram: Pearson estimation error |ΔPearson| (lower rank = better calibrated). KS-type methods are worst-calibrated; k-means/k-medoids-shuffle lead; the mirror structure is preserved.

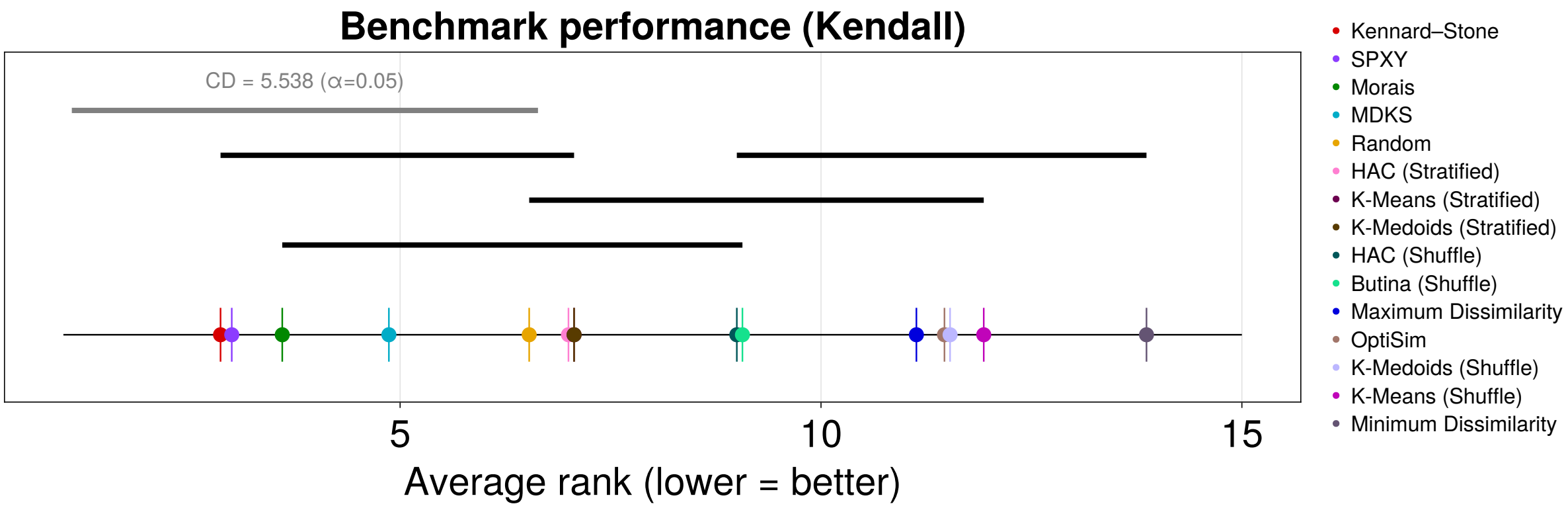

Figure 24: CD diagram: Kendall τ benchmark performance. Family ordering mirrors Spearman and Pearson: KS-type lead, diversity last, cluster-shuffle intermediate.

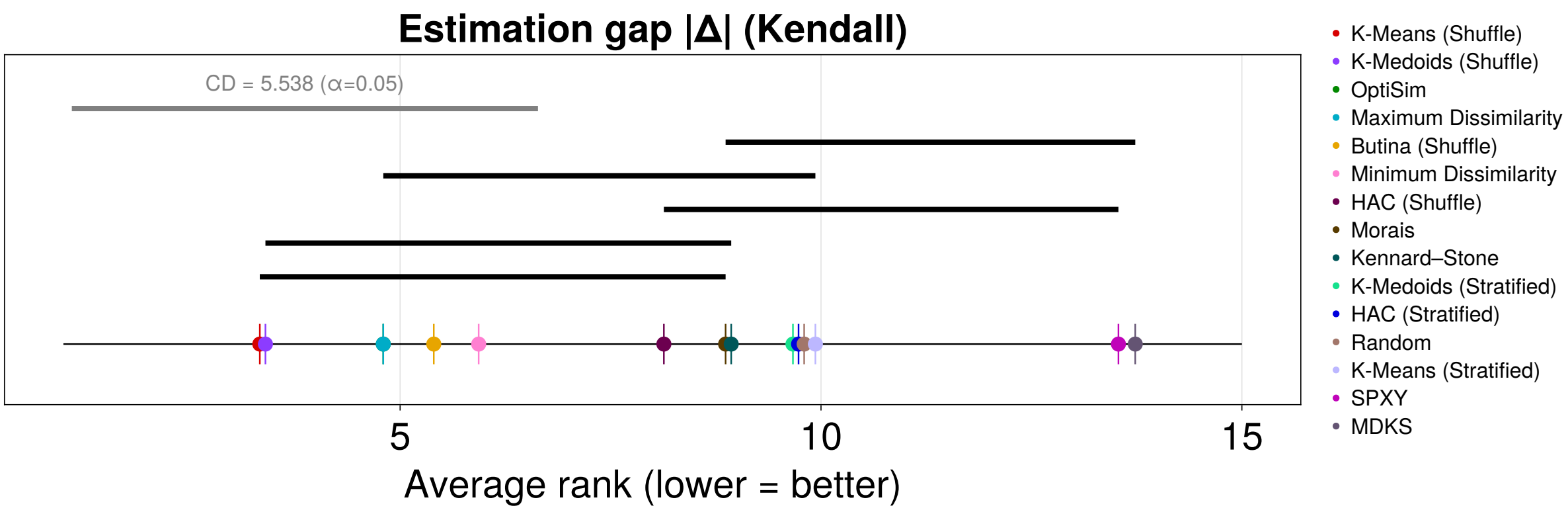

Figure 25: CD diagram: Kendall τ estimation error |ΔKendall|. Calibration ordering mirrors Spearman: k-means/k-medoids-shuffle best calibrated; KS-type worst.

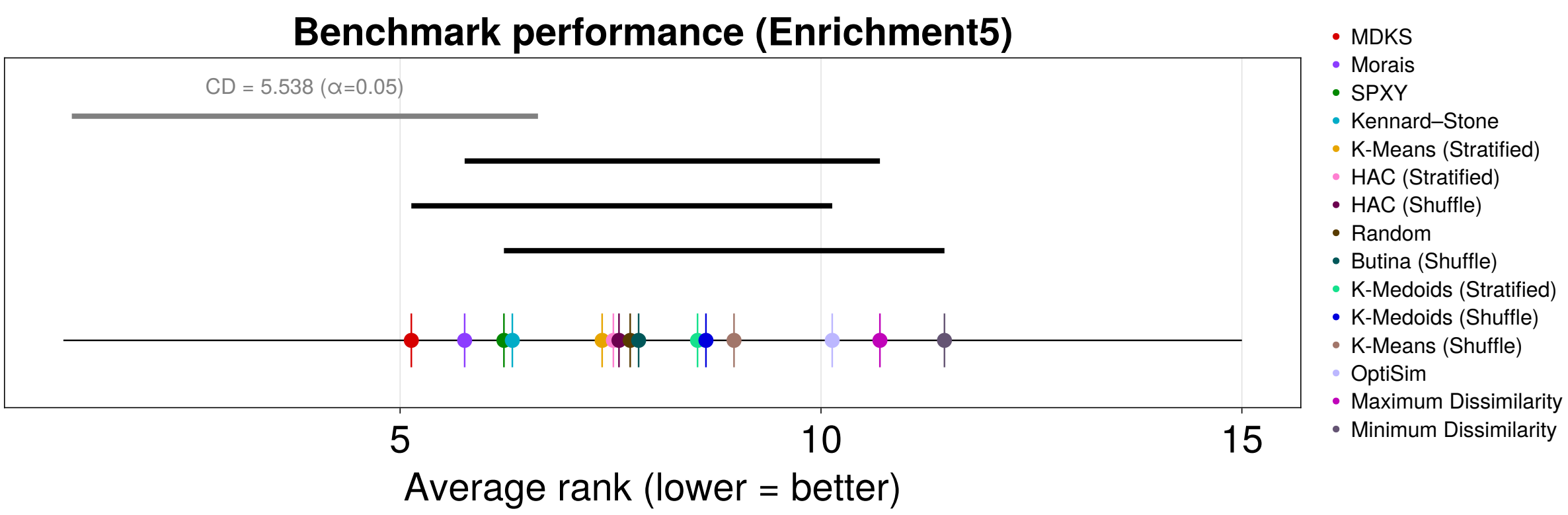

Figure 26: CD diagram: Enrichment Factor at 5% (EF-5%) benchmark performance (lower rank = higher EF-5% = better). The three-tier structure persists for enrichment: KS-type methods achieve the highest early-enrichment rankings, diversity-based methods the lowest.

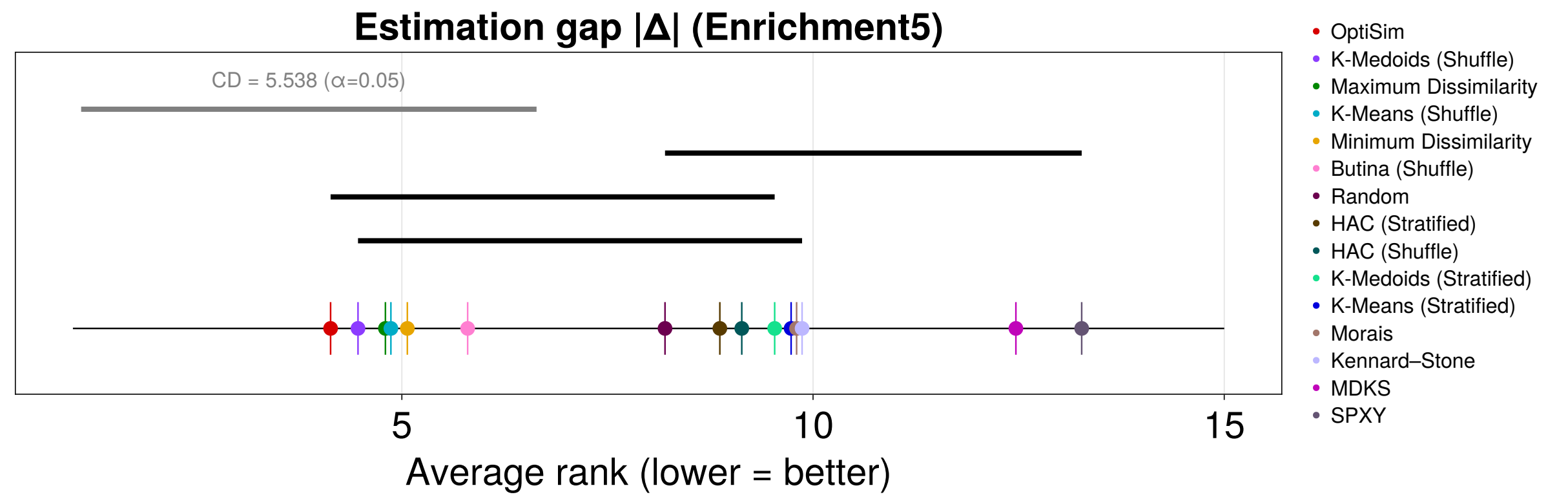

Figure 27: CD diagram: EF-5% estimation error |ΔEF-5%| (lower rank = better calibrated). KS-type methods produce the most optimistic EF-5% estimates; k-means/k-medoids-shuffle are best calibrated.

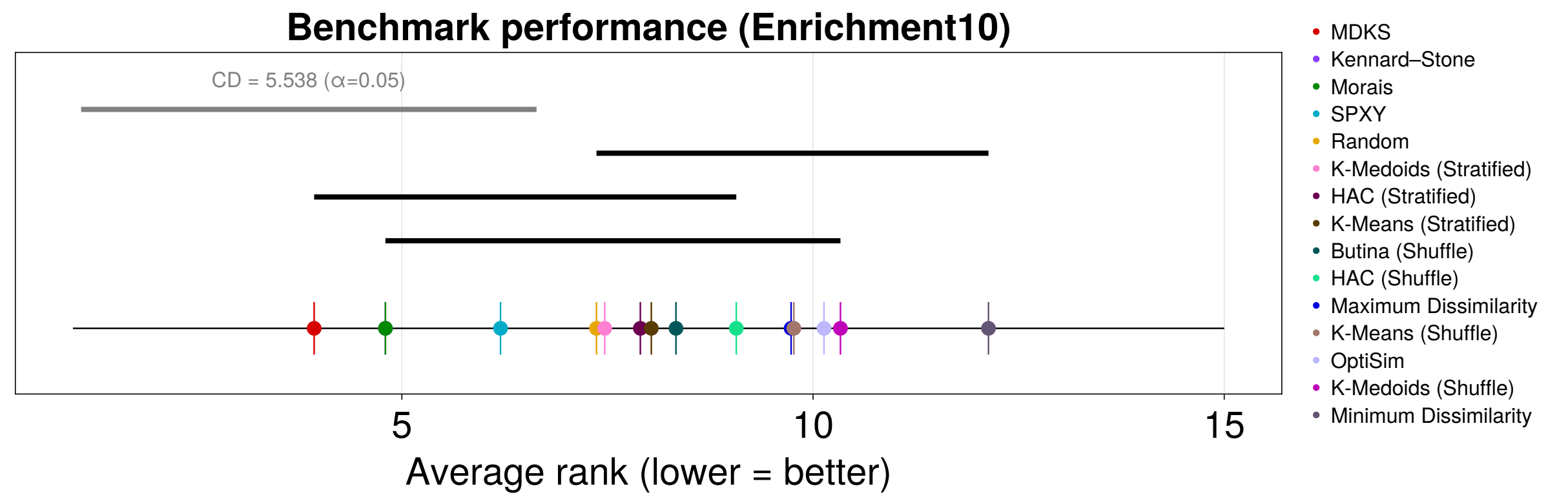

Figure 28: CD diagram: Enrichment Factor at 10% (EF-10%) benchmark performance. Family ordering mirrors EF-5%.

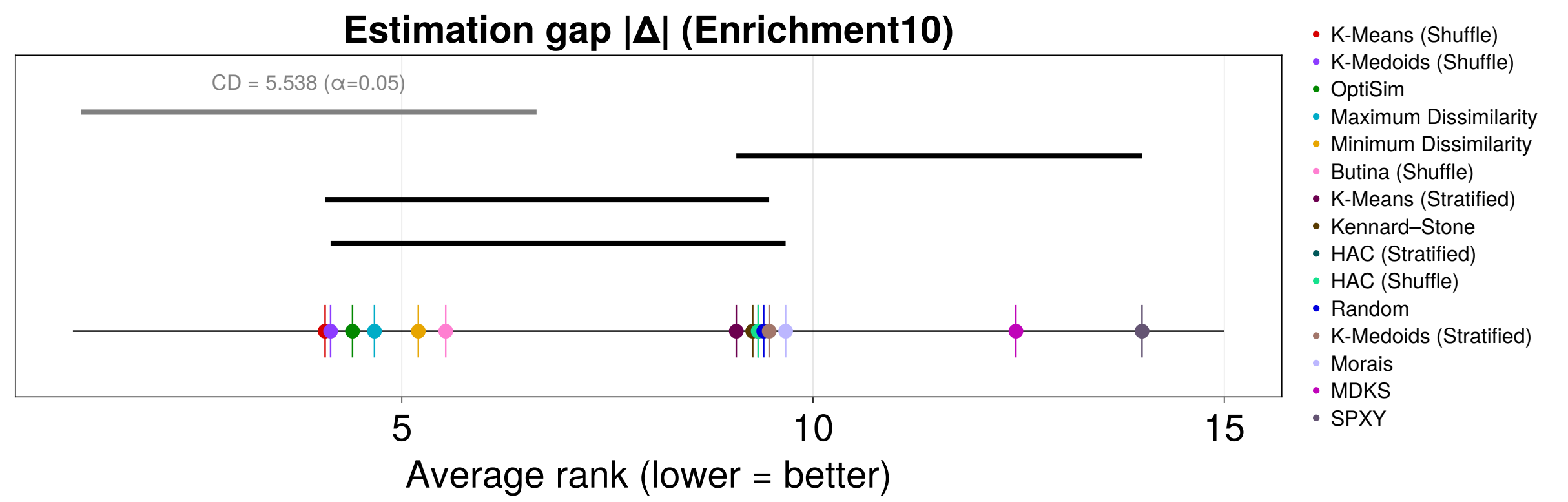

Figure 29: CD diagram: EF-10% estimation error |ΔEF-10%|. Calibration ordering mirrors EF-5%: KS-type worst-calibrated, cluster-shuffle best.

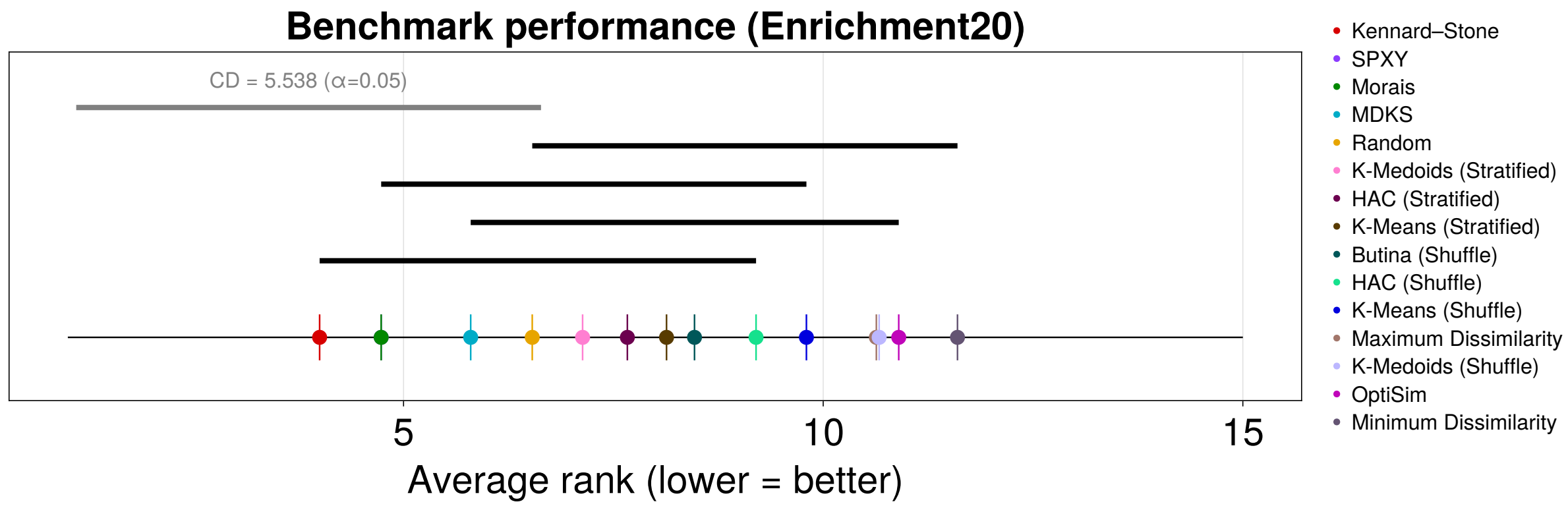

Figure 30: CD diagram: Enrichment Factor at 20% (EF-20%) benchmark performance. Family ordering is consistent with EF-5% and EF-10%.

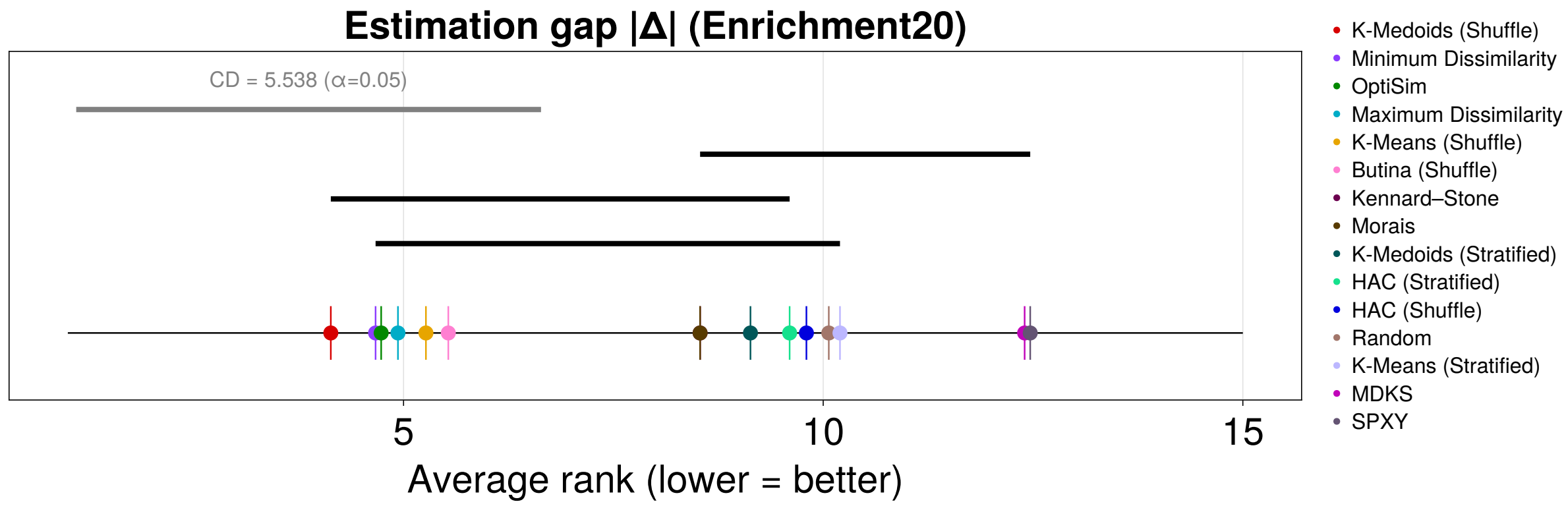

Figure 31: CD diagram: EF-20% estimation error |ΔEF-20%|. KS-type methods are worst-calibrated; cluster-shuffle methods best calibrated.

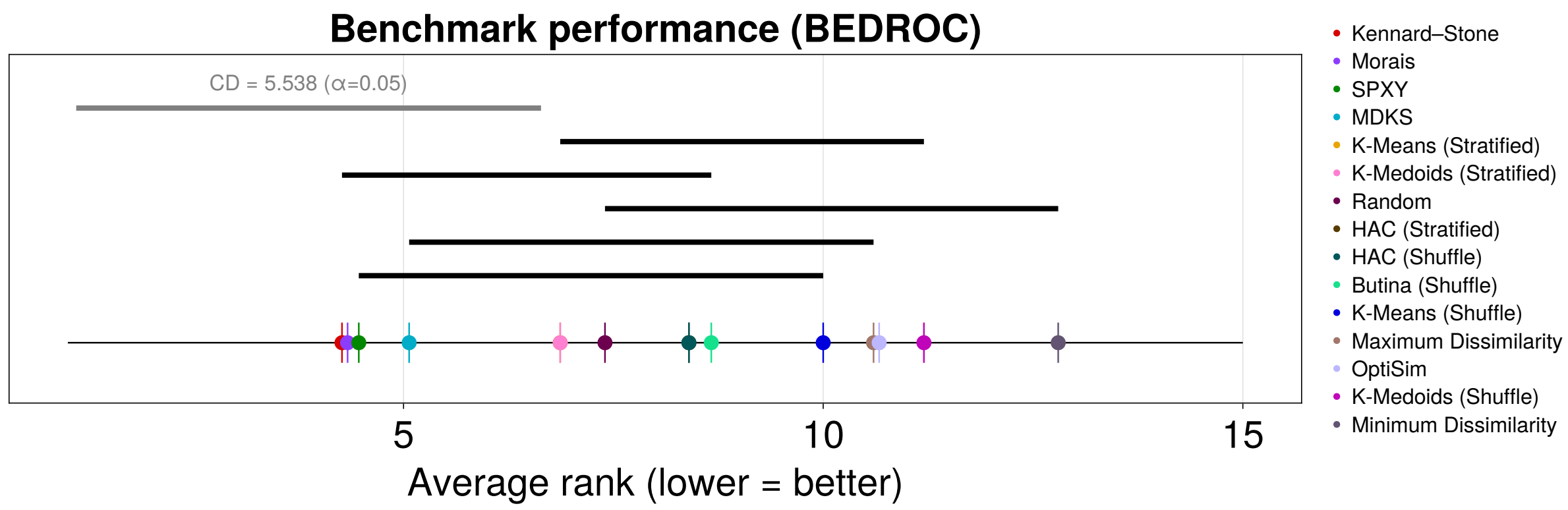

Figure 32: CD diagram: BEDROC benchmark performance (lower rank = higher BEDROC = better). The three-tier family structure is preserved for BEDROC, confirming that the KS-type advantage is not confined to aggregate measures but extends to early-enrichment scoring sensitive to the top-ranked actives.

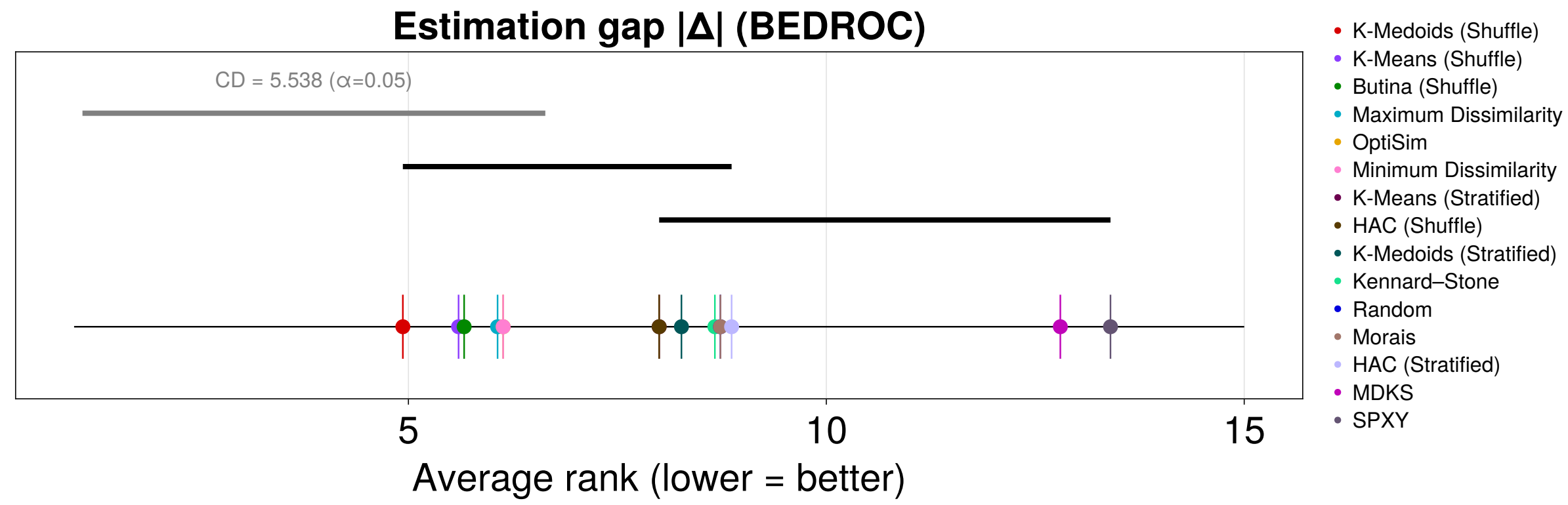

Figure 33: CD diagram: BEDROC estimation error |ΔBEDROC| (lower rank = better calibrated). KS-type methods produce optimistic BEDROC estimates; k-means/k-medoids-shuffle are best calibrated. The inversion mirrors all other measures.

above the median). The two agree to within ≈0.01 on every splitter for both quantities, and the performance–estimation inversion is preserved (rank correlation $\rho \approx -0.67$ for the fixed model vs −0.71 for the selected model). The optimism is therefore a property of the split geometry, not of the model or of hyperparameter tuning.

Splitter	Benchmark ρ (fixed)	Benchmark ρ (selected)	Bias Δ (fixed)	Bias Δ (selected)
Kennard–Stone	0.631	0.626	+0.176	+0.177
SPXY	0.631	0.631	+0.250	+0.247
MDKS	0.627	0.625	+0.209	+0.211
Morais	0.630	0.627	+0.180	+0.181
K-Means (Shuffle)	0.613	0.612	+0.064	+0.061
K-Medoids (Shuffle)	0.614	0.612	+0.071	+0.070
HAC (Shuffle)	0.619	0.617	+0.170	+0.172
Butina (Shuffle)	0.620	0.620	+0.168	+0.166
K-Means (Stratified)	0.623	0.622	+0.197	+0.197
K-Medoids (Stratified)	0.623	0.622	+0.195	+0.195
HAC (Stratified)	0.624	0.622	+0.195	+0.195
OptiSim	0.578	0.576	+0.159	+0.158
Maximum Dissimilarity	0.577	0.575	+0.159	+0.158
Minimum Dissimilarity	0.568	0.570	+0.166	+0.164
Random	0.624	0.622	+0.196	+0.198

Table S2. Hyperparameter search spaces for the five model classes in the model-selection experiment. All searches use TPE (Tree-structured Parzen Estimator) with the successive-halving schedule described in Methods. LogUniform(a, b) = log-uniform distribution on [a, b]; Uniform(a, b) = uniform on [a, b]; QuantUniform(a, b, q) = uniform on [a, b] rounded to the nearest multiple of q.

Model	Hyperparameter	Distribution / Range	Notes
**LightGBM**	`num_iterations`	QuantUniform(100, 500, step=100)	Number of boosting rounds
	`learning_rate`	LogUniform(0.005, 0.3)	Shrinkage rate
	`num_leaves`	QuantUniform(15, 255, step=1)	Max leaves per tree
	`feature_fraction`	Uniform(0.5, 1.0)	Fraction of features per iteration
	`min_data_in_leaf`	QuantUniform(5, 100, step=5)	Minimum samples in a leaf
**Random Forest**	`n_trees`	QuantUniform(50, 500, step=50)	Number of trees
	`max_depth`	Choice(−1, 5, 10, 20)	−1 = unlimited depth
	`min_samples_leaf`	QuantUniform(1, 20, step=1)	Minimum samples per leaf
	`min_samples_split`	QuantUniform(2, 20, step=1)	Minimum samples to split a node
**KNN**	`K`	QuantUniform(1, 25, step=1)	Number of neighbors
	`weights`	Choice(Uniform, InverseDistance)	Neighbor weighting
	`metric`	Choice(Euclidean, Cityblock)	Distance metric
**SVR (RBF)**	`C`	LogUniform(0.01, 100)	Regularization strength
	γ	LogUniform(1×10^{-4}, 10)	RBF kernel bandwidth
	ε	LogUniform(1×10^{-3}, 1.0)	ε-tube half-width
**Elastic Net**	λ	LogUniform(1×10^{-4}, 10)	Overall regularization strength
	γ (L1 ratio)	Uniform(0, 1)	0 = Ridge, 1 = Lasso

Table S3. Model-selection quality measures and effect sizes for all fifteen splitting strategies. Hit rate = fraction of CV folds in which the internally selected model is also the externally best-performing model (higher is better). Selection advantage = external benchmark Spearman ρ of the internally selected model minus that of the best runner-up (positive = correct selection). Both statistics are computed over 15 datasets; mean and SD are across datasets. Cohen's d is computed relative to SPXY (the top-ranked splitter on both measures) using a pooled-SD denominator; positive d indicates SPXY outperforms the listed splitter. Splitters ordered by mean hit rate.

Splitter	Hit rate mean (SD)	Hit rate median	Adv. mean (SD)	Adv. median	*d* hit rate vs SPXY	*d* adv. vs SPXY
SPXY	0.595 (0.317)	0.720	+0.0089 (0.0361)	+0.006	0.00	0.00
Butina (Shuffle)	0.587 (0.330)	0.680	+0.0036 (0.0365)	+0.006	0.02	0.15
HAC (Stratified)	0.581 (0.312)	0.600	+0.0059 (0.0289)	+0.006	0.04	0.09
K-Medoids (Stratified)	0.560 (0.295)	0.560	+0.0042 (0.0310)	+0.001	0.11	0.14
HAC (Shuffle)	0.557 (0.301)	0.560	+0.0026 (0.0352)	+0.004	0.12	0.18
K-Means (Stratified)	0.552 (0.312)	0.560	+0.0015 (0.0282)	−0.004	0.14	0.23

Splitter	Hit rate mean (SD)	Hit rate median	Adv. mean (SD)	Adv. median	*d* hit rate vs SPXY	*d* adv. vs SPXY
Kennard–Stone	0.547 (0.318)	0.520	+0.0020 (0.0350)	+0.003	0.15	0.19
Random	0.547 (0.344)	0.560	+0.0022 (0.0338)	+0.006	0.15	0.19
Morais	0.547 (0.347)	0.640	+0.0056 (0.0391)	+0.006	0.14	0.09
Maximum Dissimilarity	0.536 (0.272)	0.560	−0.0040 (0.0367)	+0.004	0.20	0.36
MDKS	0.536 (0.310)	0.600	+0.0008 (0.0345)	+0.004	0.19	0.23
K-Medoids (Shuffle)	0.533 (0.308)	0.600	+0.0032 (0.0367)	+0.006	0.20	0.16
OptiSim	0.509 (0.323)	0.560	−0.0024 (0.0415)	+0.007	0.27	0.29
K-Means (Shuffle)	0.496 (0.306)	0.560	+0.0006 (0.0325)	+0.010	0.32	0.24
Minimum Dissimilarity	0.480 (0.295)	0.440	−0.0080 (0.0384)	−0.005	0.37	0.46

Multicriteria Decision Analysis

Table S4. TOPSIS mean ranks (out of 15; lower is better) of each splitting strategy under the three criterion sets (benchmark performance, estimation credibility, and their equal-weight combination), averaged within each dataset and then across the fifteen datasets. Rows are ordered by the combined rank. This is the full ranking summarized in the main-text Multicriteria Decision Analysis section.

Splitter	Family	Performance	Credibility	Combined
K-Medoids (Shuffle)	Clustering (Shuffle)	11.3	3.8	3.8
K-Means (Shuffle)	Clustering (Shuffle)	11.2	4.0	3.9
Butina (Shuffle)	Clustering (Shuffle)	8.6	5.3	4.9
Maximum Dissimilarity	Diversity	12.3	5.3	6.6
Minimum Dissimilarity	Diversity	13.1	5.3	6.6
OptiSim	Diversity	11.9	5.5	6.8
HAC (Shuffle)	Clustering (Shuffle)	8.5	8.3	7.9
Morais	Kennard–Stone	3.6	8.7	8.1
Kennard–Stone	Kennard–Stone	3.8	8.7	8.5
K-Means (Stratified)	Clustering (Stratified)	6.3	9.1	8.5
Random	Random	6.5	8.9	8.7
HAC (Stratified)	Clustering (Stratified)	6.1	9.6	9.0
K-Medoids (Stratified)	Clustering (Stratified)	6.9	9.5	9.2
SPXY	Kennard–Stone	4.3	14.1	13.6
MDKS	Kennard–Stone	5.7	13.9	13.7

Table S5. MOORA (ratio system) mean ranks (out of 15; lower is better) for the same three criterion sets, computed and aggregated identically to Table S4. The ordering matches TOPSIS closely (Spearman $\rho = 0.98$ between the two methods' combined ranks): the three best-ranked strategies are identical and the two worst are the same pair (SPXY and MDKS), confirming that the overall ranking is not an artefact of the choice of MCDA method.

Splitter	Family	Performance	Credibility	Combined
K-Medoids (Shuffle)	Clustering (Shuffle)	11.4	3.6	3.6

Splitter	Family	Performance	Credibility	Combined
K-Means (Shuffle)	Clustering (Shuffle)	11.5	3.8	3.9
Butina (Shuffle)	Clustering (Shuffle)	9.1	5.1	5.0
Maximum Dissimilarity	Diversity	12.2	5.6	6.2
OptiSim	Diversity	11.8	5.6	6.3
Minimum Dissimilarity	Diversity	13.1	5.6	6.9
Morais	Kennard–Stone	3.3	8.5	7.8
HAC (Shuffle)	Clustering (Shuffle)	8.5	8.2	7.9
Kennard–Stone	Kennard–Stone	2.7	8.4	8.1
HAC (Stratified)	Clustering (Stratified)	7.1	9.5	9.0
K-Means (Stratified)	Clustering (Stratified)	6.5	9.1	9.1
Random	Random	6.7	9.5	9.1
K-Medoids (Stratified)	Clustering (Stratified)	6.9	9.3	9.2
MDKS	Kennard–Stone	5.3	13.9	13.9
SPXY	Kennard–Stone	3.9	14.3	14.1

References
